# Aging-related vulnerability in dopamine–glutamate neurons weakens entorhinal dopamine signaling and underlies novelty discrimination deficits

**DOI:** 10.21203/rs.3.rs-9321992/v1

**Published:** 2026-04-26

**Authors:** Jacquelyn N. Tomaio, Yifei Li, Omar Ghazy, Gillian H. Pavia, Sixtine Fleury, Alexandra Bilder, Jordan Nacimba, Isayana Castillo, William Hilaire, Aaliyah Balogun, Lakshman Abhilash, Yoon Seok Kim, Charu Ramakrishnan, Lief E Fenno, Karl Deisseroth, Susana Mingote

**Affiliations:** 1 Advanced Science Research Center at the Graduate Center, CUNY, New York, NY 10031, USA; Graduate Program in Biology, CUNY Graduate Center, New York, NY 10016, USA; 2 Department of Bioengineering, Stanford University, Stanford, CA 94305, USA; 3 Department of Psychiatry and Behavioral Sciences, Stanford University, Stanford, CA 94305, USA; Department of Bioengineering, Stanford University, Stanford, CA 94305, USA; 4 Department of Psychiatry and Behavioral Sciences, Stanford University, Stanford, CA 94305, USA; Department of Bioengineering, Stanford University, Stanford, CA 94305, USA; Howard Hughes Medical Institute, Stanford University, Stanford, CA 94305, USA

## Abstract

The lateral entorhinal cortex supports novelty detection and episodic memory and is highly vulnerable to aging. Dopamine conveys novelty signals in this region, but how aging alters this input is unknown. Using viral strategies to distinguish ventral tegmental area dopamine neurons with or without glutamate co-release, we show that co-releasing neurons comprise about 30% of dopamine neurons yet provide nearly all dopaminergic input to the lateral entorhinal cortex. In aged mice, these axons show reduced markers of dopamine synthesis and diminished dopamine release during high-frequency activity, whereas glutamate-related markers are reduced to a lesser extent. Stimulation of lateral entorhinal dopaminergic axons during novelty exploration restores novelty discrimination in aged mice. Together, these findings identify the age-related vulnerability of dopamine-glutamate neurons as a circuit-specific mechanism that impairs entorhinal dopamine signaling and underlies to deficits in novelty discrimination.

## Introduction

Across species, aging is often accompanied by a gradual decline in cognitive abilities, with episodic memory among the most consistently affected domains^1, 2^. Episodic memory enables the recollection of specific events within their spatial and temporal context, and its deterioration can make daily experiences more difficult to interpret and navigate^3^. Older individuals show increased difficulty distinguishing novel from familiar experiences, along with greater susceptibility to interference and false memories^4–10^. Age-related behavioral impairments are thought to reflect deficits in mnemonic discrimination arising from impaired pattern separation. This process transforms similar inputs into distinct, non-overlapping neural representations, thereby minimizing interference during memory encoding^2, 11, 12^. When this process is impaired, older individuals and aged rodents are more likely to misclassify novel items as familiar^4, 9, 10^.

Age-related brain changes emerge in the absence of overt neurodegeneration and reflect subtle circuit-level dysfunction, particularly within medial temporal lobe networks that support memory^13–16^. Within this network, the lateral entorhinal cortex (LEC), a major input to the hippocampus, emerges as one of the earliest and most vulnerable regions in aging and preclinical Alzheimer’s disease^14, 17–20^. The LEC supports novelty detection, item-context integration, and associative memory^21–26^, and reduced LEC engagement in older adults has been linked to poorer novel object discrimination and pattern separation^27^. However, the afferent inputs and neuromodulatory signals that underlie the age-related attenuation of LEC novelty responses remain unknown.

Aging is accompanied by a decline in dopaminergic neuromodulation^28^, which is not uniform across the forebrain. Human imaging studies indicate that the decline of dopaminergic markers follows region-specific trajectories across the adult lifespan^29–31^. Together with evidence that VTA dopamine neurons comprise distinct subpopulations that innervate different targets^32–38^, these findings raise the possibility that specific dopaminergic subpopulations are differentially vulnerable to aging.

Among VTA dopamine neuron subpopulations, dopamine-glutamate (DA–GLU) neurons are of particular interest. VTA neurons can be subdivided by their ability to co-release additional neurotransmitters^39, 40^, and DA–GLU neurons represent a distinct subgroup defined by glutamate co-transmission^41–45^. These neurons depend on vesicular glutamate transporter 2 (VGLUT2) for glutamate packaging and release. They also respond strongly to salient events, such as novelty, and contribute to behavioral flexibility^40, 42, 46–50^. Dopamine neurons, project to the LEC^51, 52^, including DA–GLU neurons^36, 53^, and recent work demonstrated that dopaminergic projections to the LEC support associative memory, in part by encoding cue novelty^54^. Together, these observations point to DA–GLU neurons as a key regulator of novelty-related processing in this region. Co-transmitting dopamine neurons are increasingly recognized as vulnerable to aging, as aged mice show reduced tyrosine hydroxylase (TH) mRNA expression in the VTA, reflecting lower expression of the enzyme required for dopamine synthesis, together with reduced VGLUT2 mRNA expression^55^.

Here, we determined how aging affects VTA dopamine neuron subpopulations and their projections to the LEC, using intersectional strategies to label defined dopamine subpopulations based on co-transmission profiles. We find an age-related vulnerability in DA–GLU neurons projecting to LEC that weakens dopamine release and is involved in deficits in novelty discrimination.

## Results

### Labeling Subpopulations of Dopamine Neurons based on their Glutamate Co-Transmission Profile

**Fig. 1 |:**
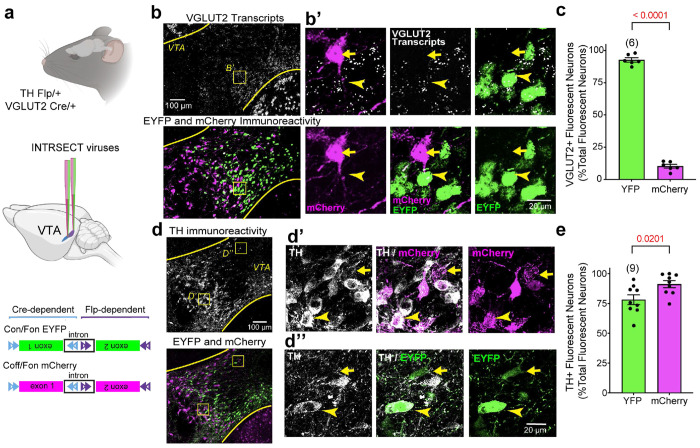
Intersectional labeling distinguishes DA–GLU and DA-only neurons in the VTA using INTRSECT viruses in young mice. **a**, Experimental schematic for double transgenic TH-Flp/+;VGLUT2-Cre/+ mice and INTRSECT constructs: Con/Fon virus drives EYFP in neurons that express both Flp and Cre (DA–GLU, shown in green) and Coff/Fon virus drives mCherry in neurons that express Flp but not Cre (DA-only, shown in magenta); schematic adapted from Fenno et al., 2020. **b**, VTA images combining VGLUT2 mRNA *in situ* (top) with EYFP and mCherry immunostaining (bottom); Inset b′ shows EYFP+ somata containing VGLUT2 transcripts (arrowheads) and mCherry+ somata not (arrows). **c**, Quantification of fluorescent neuron subtypes co-expressing VGLUT2 mRNA and EYFP (green) or VGLUT2 mRNA and mCherry (magenta), expressed as the percentage of total fluorescently labeled neurons. EYFP+ neurons show high VGLUT2 co-expression (Welch’s test, t=37.90). **d**, Confocal images of VTA sections showing fluorescent labeling of TH+ (top), and EYFP+ and mCherry+ neurons (bottom); Arrowheads in inset d’ indicate colocalization of TH and mCherry immunoreactivity; the arrow marks a rare cell that is mCherry+ but lacks TH immunoreactivity. Arrowheads in inset d′ indicate colocalization of TH and EYFP immunoreactivity; arrows mark the few EYFP+ neurons that do not express TH. **e**, Quantification of fluorescent neuron subtypes co-expressing TH protein and EYP (green) or TH protein and mCherry (magenta), expressed as the percentage of total fluorescently labeled neurons. The specificity is higher for mCherry expression (Welch’s test, t=2.612). Bar graphs show mean percentages ± SEM; number of mice shown in parentheses; *p* values shown above brackets.

To distinguish dopamine neurons that co-release glutamate (DA–GLU) from those that release only DA (DA-only), we used an INTRSECT 2.0 intersectional viral approach^56^ in double-transgenic TH-Flp/+::VGLUT2-Cre/+ mice (Fig. 1a, **top**). We injected a mixture of two INTRSECT viruses unilaterally into the middle VTA and the lateral VTA/substantia nigra pars compacta to increase the chances of transfecting all VTA dopamine neurons (Fig. 1a, **middle**). Cre-on/Flp-on (Con/Fon) virus labels neurons that co-express Flp and Cre (DA–GLU) with yellow fluorescent protein (EYFP+), whereas Cre-off/Flp-on (Coff/Fon) virus labels Flp+ neurons lacking Cre (DA-only) with mCherry fluorescent protein (mCherry+) (Fig. 1a, **bottom**).

We validated that INTRSECT expression required both recombinases (Flp and Cre) by injecting the same viral mixture into young TH-Flp or VGLUT2-Cre mice. EYFP was absent in both single-transgenic lines, confirming that Con/Fon virus expression requires both recombinases (**Extended Fig. 1a**). In contrast, mCherry was robust in TH-Flp mice (including medial VTA regions enriched in DA–GLU neurons) but absent in VGLUT2-cre mice, consistent with Coff/Fon virus activation by Flp and suppression by Cre (**Extended Fig. 1b**).

To assess glutamatergic identity and specificity, we combined VGLUT2 *in situ* hybridization with EYFP and mCherry immunohistochemistry. Confocal images (Fig. 1b) and volume renderings (**Extended Fig. 1c**) showed that 93% ± 1.6% of EYFP+ neurons contained VGLUT2 puncta, confirming successful targeting of DA–GLU neurons. mCherry+ neurons showed minimal VGLUT2 signal, although 10% ± 1.4% displayed a single VGLUT2 punctum (Fig. 1c; **Extended Fig. 1d**).

To evaluate dopaminergic specificity, we quantified co-localization with TH immunostaining. Overall, 91% ± 2.9% of mCherry+ neurons and 78% ± 4.1% of EYFP+ neurons were TH+ (Fig. 1d,e). The EYFP+ / TH– fraction may reflect a previously described subset of VTA neurons that express TH mRNA but have undetectable TH protein^57^. Given that VGLUT2 signal is present in most EYFP+ neurons, these EYFP+ / TH– cells could represent glutamatergic-only neurons with ectopic EYFP expression.

Together, these data show that the INTRSECT strategy robustly labels DA–GLU neurons with EYFP and DA-only neurons with mCherry, enabling population-specific analyses of VTA circuits and their vulnerability to aging.

### Distribution of Subpopulations of Dopamine Neurons in the VTA and LEC in Young Mice

**Fig. 2 |:**
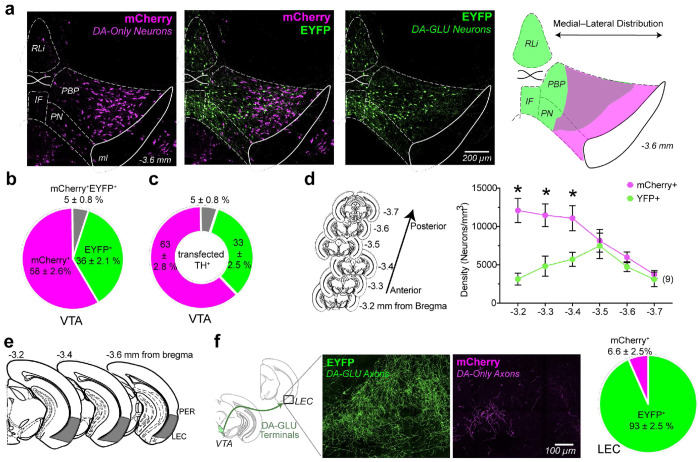
DA–GLU and DA-only neurons exhibit distinct spatial organization in the VTA and differentially innervate the LEC in young mice. **a**, Confocal photomicrographs showing the distribution of INTRSECT labeled VTA neurons expressing EYFP (DA–GLU, green), mCherry (DA-only, magenta), and merge (middle); schematic of the medial to lateral subtype distribution on the right. **b**, Proportion of EYFP+ (green), mCherry+ (magenta), and co-localized EYFP+/ mCherry+ (gray) neurons in the VTA as a percentage of all INTRSECT-labeled neurons. **c**, Proportion of EYFP+/TH+ (green), mCherry+/TH+ (magenta), and EYFP+/ mCherry+/TH+ (gray) neurons in the VTA as a percentage of all TH+ INTRSECT-labeled neurons. **d**, Schematic of coronal brain sections along the anterior–posterior VTA axis (‒3.2 to ‒3.7 mm from bregma) used for quantification (left); Line plot showing the density (neurons/mm^3^) of mCherry+ and EYFP+ neurons across the anterior–posterior VTA axis in mm from bregma (two-way repeated measures ANOVA showed a significant interaction: *F*(5, 40) = 5.58, *p* < .001; large effect size: ηp^2^ = .24; *significant Post-hoc Bonferroni comparisons, p < 0.05). **e**, Schematic of coronal brain sections from ‒3.2 mm to ‒3.6 mm relative to bregma highlighting the LEC (gray) below perirhinal cortex (PER). **f**, Schematic of VTA DA–GLU projections to the LEC (left). Representative confocal images of DA–GLU axons (EYFP+, green) and DA-only axons (mCherry+, magenta) in the LEC (middle). Pie chart shows the proportion of fluorescent axons in the LEC that are EYFP+ (green) versus mCherry+ (magenta) (right). All data shows mean density ± SEM; number of mice shown in parentheses. *IF*, interfascicular nucleus; *ml*, medial lemniscus; *PBP*, parabrachial pigmented nucleus;* PN*, paranigral nucleus; *RLi*, rostral linear nucleus.

DA neuron subpopulations show distinct prevalence and spatial distribution patterns within the VTA (Fig. 2a–c). We found that 36% of all fluorescently labeled cells in the VTA were EYFP+, and when limiting the analysis to TH+ neurons, 33% of transfected TH+ VTA neurons were EYFP+ DA–GLU neurons (Fig. 2c). This proportion is consistent with prior reports in mice, which estimate DA–GLU neurons comprise 20–30% of the VTA DA population using intersectional genetic labeling or multiplex *in-situ* hybridization^36, 40, 42, 57, 58^. We observed no differences between males (n=5) and females (n=4); therefore, the data were combined (**Extended Fig. 2a-c**).

DA–GLU and DA-only neurons also differ in their anatomical distribution. EYFP+ DA–GLU neurons are enriched in the interfascicular nucleus (IF), rostral linear nucleus (RLi), medial paranigral nucleus (PN), and medial parabrachial pigmented nucleus (PBP) of the VTA^58^. Along the anterior–posterior axis (−3.2 to −3.7 mm from bregma), DA–GLU neurons were predominantly located in the middle portion of this range, whereas DA-only neurons were more concentrated in the anterior VTA (Fig. 2d).

When examining dopaminergic projections to the LEC, we found that 93% of labeled axons were EYFP+ (Fig. 2e,f), indicating that they originated from DA–GLU neurons. This was a surprising and novel finding, given that DA–GLU neurons comprise only a minority of VTA dopamine neurons (33%). Thus, although this subpopulation represents only a small fraction of dopaminergic neurons in the VTA, it gives rise to nearly all dopaminergic input to the LEC. Together with our previous functional connectome study showing robust glutamatergic input from VTA dopamine neurons to LEC neurons^53^, these data support the conclusion that LEC-projecting dopamine neurons arise predominantly from a selectively defined neuronal subpopulation.

In summary, intersectional strategies reveal that DA–GLU neurons make up roughly one-third of VTA DA neurons, are concentrated in the medial portion of VTA, and provide the majority of DAergic innervation to the LEC.

### Effects of Aging on the Density of Dopamine Neuron Subpopulations within the VTA

Distinct dopaminergic circuits are not equally affected by aging; some projection-defined populations appear to be more vulnerable than others and may show dysfunction earlier in the aging process^29, 31, 59, 60^. Because DA-only and DA–GLU subpopulations innervate distinct brain regions^40^, we investigated whether aging differentially impacts these specific subtypes.

**Fig. 3 |:**
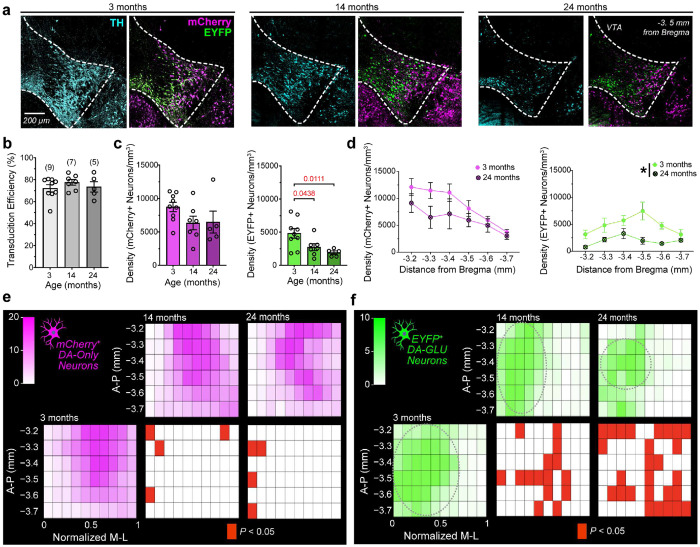
Aging alters the density and spatial distribution of dopamine neuron subpopulations in the VTA . **a,** Representative unilateral coronal sections through the VTA at approximately ‒3.5 mm from bregma in 3- (left), 14- (middle), and 24-month-old (right) mice, showing fluorescent labeling of TH+ (blue), EYFP+ (green), and mCherry+ (magenta) neurons. **b,** Bar graph showing the transfection rate, calculated as the percentage of TH+ neurons that co-expressed both EYFP and mCherry immunofluorescence relative to the total number of TH+ VTA neurons, across age groups (one-way ANOVA, F(2,18)=8.271, *p*=0.4937). **c,** Bar graph showing mCherry+ (left) and EYFP+ (right) neuron density across age groups (mCherry+ neurons: no age effect, one-way ANOVA, F(2,18)=1.95, p=0.4210; moderate effect size, ηp^2^=0.17; EYFP+ neurons: one-way ANOVA, F(2,18)=5.81, p=0.0113; large effect size, ηp^2^ = 0.39; Dunnett’s post hoc test versus 3 months).** d,** Line graphs showing the density of mCherry+ (top) and EYFP+ (bottom) neurons across six anterior-posterior VTA levels (‒3.2 to ‒3.7 mm from bregma) in 3- and 24-month-old mice. mCherry+ neurons: two-way RM ANOVA, main effect of distance from bregma, F(5,60)=8.04, p<0.0001; large effect size, ηp^2^=0.455; no main effect of age, p=0.0727, or interaction, p=0.514. EYFP+ neurons: two-way RM ANOVA, main effect of age, F(1,12)=8.29, *p*=0.0138; large effect size, ηp^2^=0.11; main effect of distance from bregma, F(5,60)=2.81, p=0.0242; large effect size, ηp^2^=0.25; no significant interaction, *p*=0.2680.** e,** Two-dimensional VTA density map showing the distribution of mCherry+ (DA-only) cells across anterior-posterior and normalized medial-lateral coordinates. Red tiles indicate bins that differed from the 3-month group (one-tailed t-test vs the 3-month mean) after Benjamini–Hochberg correction to control the false discovery rate at 5%. * = age effect. **f,** Same as in e, but for EYFP+ (DA–GLU) cells. * = age effect. Grey dashed circles identify the hotspots of DA–GLU neurons, which decrease with age. Data as mean ± SEM. Exact *p* values for post hoc comparisons are shown above the graphs, and sample sizes are indicated in parentheses for each group.

We used the same INTRSECT viral strategy described above to label DA–GLU and DA-only neurons in mice at different ages: 3 months (young, n=9), 14 months (middle, n=7), and 24 months (aged, n=5) (Fig. 3a). We injected double mutant mice with INTRSECT viruses one month before reaching the target age (above) to allow sufficient viral expression before perfusion. Age did not affect the viral transduction efficiency (~75%), quantified as the percentage of TH+ neurons in the VTA that expressed either transgene (mCherry and YFP combined) (Fig. 3a). Aging also did not alter the specificity of INTRSECT viruses in targeting DA–GLU versus DA-only neurons (**Extended Fig. 3a,b**). Having established that INTRSECT can be used to assess age-related differences, we then measured neuronal density of the labeled subpopulations. DA-only neurons showed a decreasing trend that did not reach statistical significance, whereas the density of DA–GLU neurons was significantly reduced by 14 months and declined further by 24 months, reaching an overall ~60% decrease (Fig. 3c).

We evaluated how density changes along the anterior–posterior axis by comparing 3- and 24-month-old mice (Fig.3d). Density did not vary significantly along the anterior-posterior axis for the mCherry+ DA-only subpopulation. In contrast, aged (24-month-old) mice showed lower EYFP+ DA–GLU neuron density than young (3-month-old) mice across the anterior–posterior sections sampled. However, this analysis does not account for the significant heterogeneity in distribution of the subpopulations across the medial-lateral VTA axis (Fig. 2a). To further characterize age-related changes in cell-type distribution, we generated spatial density heat maps across the anterior-posterior and medial-lateral axes (Fig.3e,f). The maximum distance of TH+ cells from the midline was used as an estimate of the VTA medial-lateral dimension. This distance was greater in young males than in young females, and greater in middle-aged compared to young mice (Extended **Fig. 3c,d**), indicating that the medial-lateral VTA size varies with sex and age, probably due to change in brain size. To enable comparisons of cell distributions across age groups, we normalized the medial-lateral axis from 0 to 1, where 1 corresponds to the maximum distance from the midline. Comparing the distribution across age groups revealed that DA–GLU neurons underwent the most pronounced spatial reorganization with aging, whereas DA-only distributions changed only modestly (Fig. 3e,f). In aged mice, the reduction in DA–GLU cell number was not uniform, but was concentrated in anterior (−3.2 mm relative to bregma) and posterior (−3.6 mm relative to bregma) VTA sections, together with broader losses across lateral regions. As a result, the hotspot of DA–GLU neurons in aged mice was smaller and restricted to a medial band at approximately −3.3 to −3.4 mm relative to bregma (grey dashed circles, Fig. 3f). Consistent with this pattern, a DA–GLU minus DA-only difference map showed clear spatial segregation in young mice (ventromedial DA–GLU enrichment and lateral DA-only enrichment) that progressively collapsed with age, yielding widespread DA-only predominance and supporting preferential vulnerability of DA–GLU neurons and region-specific loss of their spatial niches (**Extended Fig. 3e)**.

In addition to a change in INTRSECT-labeled subpopulations, we also observed a decrease in the density of TH+ neurons in both middle-aged and aged mice (**Extended Fig. 3f**). The spatial mapping revealed little regional redistribution, with few bins showing significant age differences, consistent with a broadly uniform reduction rather than focal loss. Reduced TH expression could contribute to the loss of EYFP+ labeling, because INTRSECT expression depends on TH promoter activity in addition to VGLUT2 promoter activity. However, the spatial pattern of EYFP+ changes did not mirror the TH+ map, showing that the reduction in labeling cannot be explained solely by decreased TH expression.

Overall, our data identify DA–GLU neurons as a selectively vulnerable VTA subpopulation, with INTRSECT-labeling reduced by 14 months and further diminished in aged (24 months) mice. Spatial mapping indicates that this loss is clustered rather than uniform, pointing to the possibility that specific dopaminergic pathways may be preferentially affected, given the medial– lateral topography of VTA forebrain projections^34^. Finally, the age-related spatial changes in EYFP+ DA–GLU neurons did not mirror those of TH+ cells, indicating that the reduction in labeling cannot be explained solely by decreased TH expression and raising the possibility that diminished VGLUT2 expression, together with reduced TH, may produce a “double hit” that contributes to the loss of DA–GLU labeling.

### Effects of Aging on the Density of Dopaminergic Projections to the LEC

**Fig. 4 |:**
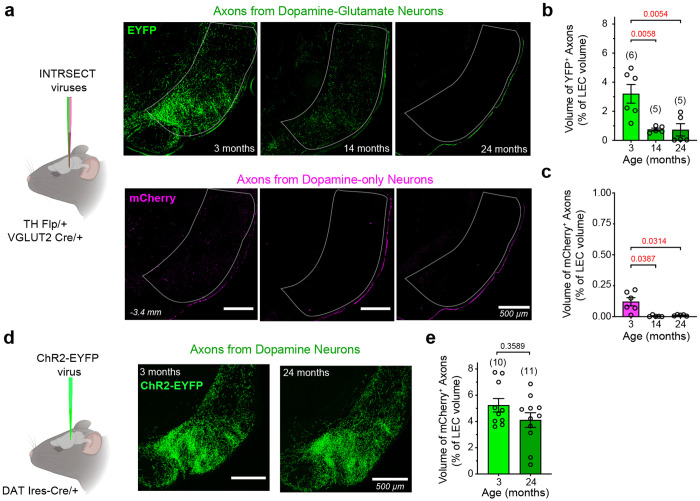
Distinct viral labeling strategies reveal an age-related reduction in EYFP+ DA–GLU axonal density in the LEC that is not attributable to axonal degeneration. **a,** Experimental schematic showing INTRSECT viruses injected into TH Flp/+ ; VGLUT2 Cre/+ mice (left), and representative images of EYFP+ DA–GLU axons (top right) and mCherry+ DA-only axons (bottom right) in the outlined LEC at 3, 14, and 24 months. **b,** Relative volume of EYFP+ DA– GLU axons, expressed as a percentage of total LEC volume, decreased with age (Kruskal–Wallis, H(2) = 9.263; large effect size, ηp^2^= 0.56). Dunn’s post hoc test showed significant reductions at 14 and 24 months relative to 3 months. **c,** Relative volume of mCherry+ dopamine-only axons, expressed as a percentage of total LEC volume, also differed by age (Kruskal–Wallis, H(2) = 9.801; large effect size, ηp^2^ = 0.60), driven by a reduction at 14 months, but not at 24 months, relative to 3 months (Dunn’s post hoc test). **d,** Experimental schematic showing ChR2-EYFP virus injected into DAT Ires-Cre/+ mice to label all dopaminergic axons (left), with representative images of ChR2-EYFP+ axons in the LEC at 3 and 24 months (right). **e,** ChR2-EYFP+ axon volume, expressed as a percentage of total LEC volume, did not differ between 3- and 24-month-old mice (two-tailed Mann–Whitney test, U = 68.5; small effect size, rank-biserial correlation, r_rb = 0.25). Data are shown as mean ± s.e.m. Exact p values are shown above the graphs (red <0.05) and sample sizes are indicated in parentheses for each group.

Since DA–GLU neurons provide the predominant dopaminergic input to the LEC, we next asked whether their age-related decline was accompanied by a corresponding reduction in INTRSECT-labeled axons within this region (Fig. 4a). To quantify terminal density, we performed 3D reconstructions of INTRSECT-labeled axons using volume-rendering software and measured their volume relative to the total volume of the LEC. We observed a marked reduction (~75%) in the density of EYFP+ DA–GLU axons in the LEC in both middle-aged and aged mice (Fig. 4b). In some aged animals, EYFP labeling was nearly absent, as illustrated in the representative photomicrograph (top right, Fig. 4a). We also examined the smaller population of mCherry+ DA-only axons in the LEC and found that their density was also significantly reduced at 14 and 24 months (Fig. 4c). In middle-aged mice, the density of labeled DA–GLU neurons in the VTA declined by ~40%, whereas INTRSECT-labeled axons in the LEC were reduced by nearly 75%. This disproportionate loss suggests that LEC dopaminergic input from DA–GLU neurons is especially vulnerable early in aging.

We further investigated why INTRSECT labeling of DA–GLU neurons and their axons in the LEC declines with age. A recent study reported that TH and VGLUT2 RNA expression in the VTA decreases in aged mice without evidence of cell loss^55^. We therefore hypothesized that reduced expression of TH or VGLUT2 may underlie the diminished INTRSECT labeling, since this viral strategy depends on the activity of both promoters. To test this, we used an alternative viral strategy in which YFP is expressed under the DAT promoter, independent of TH or VGLUT2 expression status. An AAV-FLEX-ChR2-YFP virus was injected into the VTA of DAT- Ires-Cre mice at 2 or 23 months of age to generate young (3-month-old, *n* = 10) and aged (24-month-old, *n* = 11) cohorts. With this approach, YFP+ dopaminergic axons in the LEC were clearly observed in both groups (Fig. 4d), and quantification of terminal volume revealed no age-related differences (Fig. 4e). Thus, terminal density in the LEC is largely preserved at 24 months. Taken together, these results argue against cell loss or terminal degeneration and instead suggest that the reduction in INTRSECT labeling reflects decreased TH or VGLUT2 expression with age.

### Effects of Aging on Markers of Dopamine Synthesis and Glutamate Vesicular Loading in LEC DA–GLU Axons

**Fig. 5 |:**
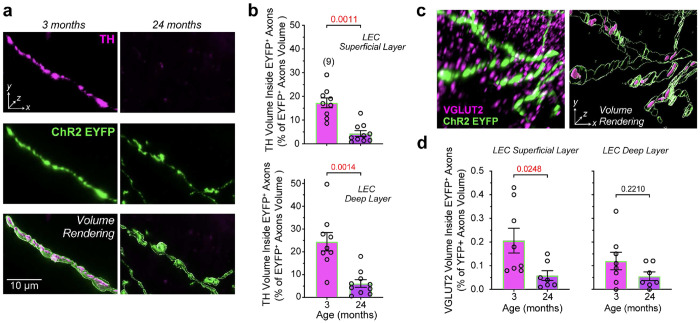
Age-related reduction of TH and VGLUT2 content in LEC-projecting dopaminergic terminals. **a,** Representative high-magnification images of axons in the LEC of 3-month-old (left) and 24-month-old (right) DAT- Ires-Cre mice injected with a ChR2-EYFP-expressing virus. Each column shows TH immunoreactivity (top, magenta), ChR2-EYFP signal (middle, green), and a volume rendering (bottom) of the merged signals from the same field of view (also see supplementary video 1 and 2). **b,** Quantification of TH signal volume within EYFP+ axons, expressed as a percentage of total EYFP+ axon volume, in the superficial (top) and deep (bottom) layers of the LEC. Aged mice exhibited a significant reduction in TH content in both layers (superficial layer: *t*(17) = 5.51, Cohen’s *d* = 2.53; deep layer: *t*(17) = 4.42, Cohen’s *d* = 2.03). **c,** Representative high-resolution image of VGLUT2 puncta (magenta) colocalized with EYFP-labeled axons (green) in the LEC, shown as a raw fluorescence image (left) and a 3D volume rendering of the same region (right). **d,** Quantification of VGLUT2 signal volume within EYFP+ axons, expressed as a percentage of total EYFP+ axon volume, in the superficial (left) and deep (right) layers of the LEC. Aged mice showed a significant reduction in VGLUT2 content in the superficial layer (two-tailed Mann–Whitney test, *U* = 8; large effect size, rank-biserial correlation, *r*_rb = -0.78), whereas no significant difference was detected in the deep layer (Welch’s two-tailed unpaired *t*-test, *t* = 1.573; moderate effect size, Hedges’ *g* = 0.73). Data as mean ± SEM. Exact *p* values above the graphs, and sample sizes are shown in parentheses for each group.

We investigated whether EYFP-labeled dopaminergic axons in the DAT-Ires-Cre mouse expressed the proteins TH and VGLUT2. To quantify TH expression, we performed volume rendering and generated surfaces of both TH and YFP immunoreactivity (Fig. 5a). TH clusters overlapping by more than 65% with EYFP labeling were considered to be inside EYFP+ axons, and the volume of TH inside axons was calculated relative to the total volume of EYFP+ axons in the LEC. We quantified the superficial and deep layers of the LEC separately, as these layers are functionally distinct^61–63^ and EYFP+ axons were denser in the superficial layer (**Extended Fig. 4a**). The expression of TH within EYFP+ axons was significantly reduced with age in both layers (Fig. 5A, B, **supplementary video 1 and 2**). However, TH immunoreactivity outside EYFP+ axons did not differ between young and aged mice (**Extended Fig. 4b**). Because noradrenergic neurons also innervate the LEC^51^, it is plausible that the TH signal not associated with EYFP+ fibers reflects noradrenergic projections.

We then assessed whether VGLUT2 expression is also reduced within EYFP-labeled axons. VGLUT2 immunolabeling appeared as densely clustered puncta in the LEC, reflecting expression in both DAergic axons and other glutamatergic inputs, with the latter providing the majority of VGLUT2. The overall volume of VGLUT2 puncta in the LEC did not change with aging (**Extended Fig. 4c**). To quantify VGLUT2 inside YFP+ axons, we acquired images with a confocal microscope equipped with Airyscan super-resolution detection^64^. Compared to conventional confocal microscopy, Airyscan employs a detector array with pixel reassignment and deconvolution, improving lateral resolution to ~120 nm and enhancing signal-to-noise, thereby allowing a more reliable visualization of densely packed puncta. VGLUT2 puncta overlapping by more than 65% with YFP labeling were considered inside YFP+ axons (Fig. 5c). Using this conservative criterion, we found that in young mice, only ~0.2 % of total VGLUT2 expression in the LEC was localized inside dopaminergic axons, confirming that the vast majority of VGLUT2 arises from non-dopaminergic inputs. As with TH, we analyzed superficial and deep layers separately, calculating the volume of VGLUT2 puncta as a percentage of total YFP+ axonal volume (Fig. 5d). In the superficial layer, VGLUT2 volume was significantly reduced in aged mice. In contrast, in the deep layer the difference between ages did not reach significance.

In conclusion, TH expression was robustly reduced within dopaminergic axons in the LEC across both superficial and deep layers. VGLUT2 expression declined with aging in a layerspecific manner, reaching significance only in the superficial LEC. Together, these findings indicate that DA–GLU neurons projecting to the LEC undergo marked age-related deterioration, with a substantial loss of dopamine synthesis capacity and a more moderate, regionally restricted decline in glutamate vesicular loading.

### Effects of Aging on Dopamine Release in the LEC

**Figure 6 |:**
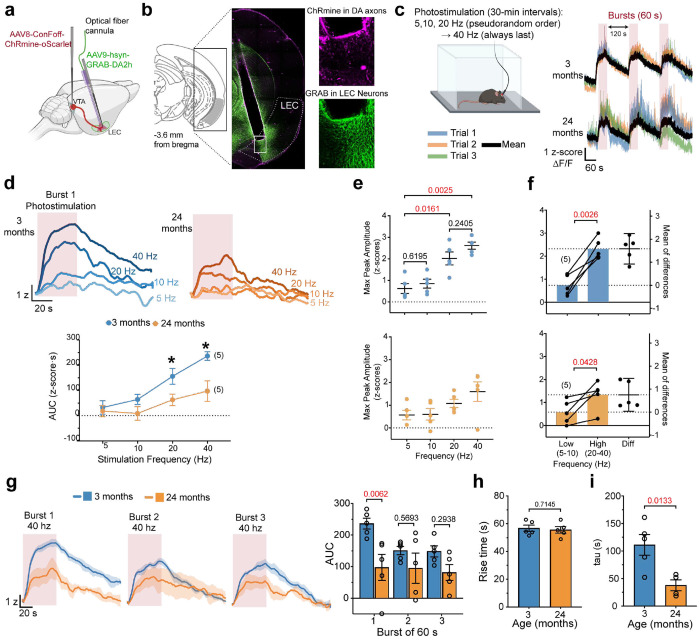
Aged mice show frequency-dependent decreases in evoked dopamine release. **a,** Schematic of the viral strategy used for fiber photometry recordings. **b,** Coronal section showing optic fiber placement in the LEC; insets show ChRmine (magenta) and GRAB^DA2h^ (green) expression. **c,** Optogenetic stimulation protocol and representative raw traces in response to 40 Hz from one young and one aged mouse. **d,** Mean traces for the first burst at each frequency in young (blue) and aged (orange) mice. The plot shows area under the curve (AUC) by age and frequency. RM ANOVA revealed a significant age × frequency interaction (F(2.278,18.22) = 4.612, p = 0.0205), a main effect of frequency (F(2.278,18.22) = 28.68, p < 0.0001) and age (F(1,8) = 5.700, p = 0.0440). Planned comparisons using one-tailed unpaired Welch’s t-tests (young > aged) with Holm–Bonferroni correction were significant at 20 Hz (t(7.31) = 2.39, p = 0.0237) and 40 Hz (t(5.37) = 3.12, p = 0.0237), with Hedges’ g = 1.36 and 1.79, respectively. **e,** Maximum peak amplitude by stimulation frequency in young (top) and aged (bottom) mice. RM ANOVA with Geisser–Greenhouse correction showed a frequency effect in young mice (F(1.863,7.452) = 25.56, p = 0.0005, ηp^2^ = 0.865) and no effect in aged mice (F(1.544, 6.174) = 4.978, p = 0.0570, ηp^2^ = 0.554). **f,** Paired estimation plots comparing low (5–10 Hz) and high (20–40 Hz) stimulation frequencies in young (top) and aged (bottom) mice. Left, paired averages for each mouse with paired t-test p values above brackets; right, paired mean difference (high minus low). **g,** Mean traces for 40 Hz stimulation across three consecutive bursts. Right, RM ANOVA showed a significant age × burst interaction (F(2,16) = 4.031, p = 0.0382), a main effect of burst (F(2,16) = 5.201, p < 0.0182), and a main effect of age (F(1,8) = 5.776, p < 0.0430). Post hoc šídíák comparisons above brackets. **h,** Rise time for the first 40 Hz burst showed no age effect (unpaired t-test, t = 0.3791). **i,** Decay time constant (τ) for the first 40 Hz burst was reduced in aged mice (unpaired t-test, t = 3.217). Data are mean ± SEM; sample sizes are shown in parentheses. P values above brackets (red>0.05).

Decreases in TH expression are typically associated with reduced dopamine release^65^; however, compensatory mechanisms are common in dopamine neurons and can preserve dopaminergic function^66^. Thus, we evaluated the impact of TH reduction on dopamine release by optogenetically stimulating dopaminergic axons in the LEC (red-shifted ChRmine) and measuring dopamine dynamics with the green dopamine sensor GRAB_DA2h_ using fiber photometry (Fig. 6a,b; **Extended Fig. 5a**).

Dopamine neurons are known to have two modes of firing, a tonic firing (4-10 Hz) and phasic firing (20-100Hz)^67–69^. Tonic firing is thought to maintain baseline dopamine tone, while phasic burst firing conveys event-related signals about novelty and salience through brief, larger dopamine transients^67, 69–72^. We reasoned that if dopamine synthesis is compromised, it would impair the maintenance of dopamine release during repeated stimulation or at higher frequencies. Thus, we stimulated dopaminergic axons with three consecutive 60-s bursts at four frequencies (5, 10, 20, and 40 Hz). The order of the 5, 10, and 20 Hz stimulation frequencies was randomly assigned. The 40 Hz stimulation was delivered at the end of the session to minimize the possibility that high-frequency–induced plasticity^73^ could affect responses at the other frequencies (Fig. 6c, **left**). For each frequency, bursts were delivered across three trials, responses were averaged, and mean traces were compared across frequencies (Fig. 6c, **right**).

To test for potential crosstalk between the red stimulation and green recording channels, we measured GRABDA2h signals in response to red light in the absence of ChRmine expression. We observed no light-evoked signal, indicating that red stimulation did not produce detectable crosstalk in the green recording channel (**Extended Fig. 5b-e**). We next examined how stimulation frequency shaped dopamine release during the first burst. In young mice, increasing stimulation frequency increased dopamine release, measured as area under the curve, consistent with prior reports in other dopaminergic targets^74–77^ (Fig. 6d). In aged mice, release also increased with frequency, but this relationship was markedly attenuated. Comparisons between age groups revealed reduced release in aged mice only at the higher frequencies, 20 and 40 Hz, whereas responses at 5 and 10 Hz were preserved. Analysis of maximum peak amplitude further showed that, in young mice, responses segregated into two frequency ranges: 5 and 10 Hz did not differ from each other, and 20 and 40 Hz did not differ from each other, whereas both high frequencies evoked larger responses than 5 Hz (Fig. 6e, **top**). A planned comparison confirmed that the average response at high frequencies (20–40 Hz) was greater than at low frequencies (5–10 Hz) (Fig. 6f, **top**). In aged mice, maximum peak amplitude did not significantly increase with stimulation frequency, although there was a trend in that direction (Fig. 6e, **bottom**). Accordingly, the separation between low- and high-frequency responses was markedly reduced and only marginally significant in aged mice (Fig. 6f, **bottom**). Together, these findings indicate that aging narrows the frequency-dependent dynamic range of dopamine release between tonic- and phasic-like activity, primarily by blunting responses to high- frequency stimulation.

In young animals, the dynamics of dopamine release across the three bursts depended on stimulation frequency (**Extended Fig. 6a,c,e,g**). At 5 Hz, repeated stimulation produced shortterm facilitation, with a progressive increase from the first to the third burst. At 10 Hz, response amplitude remained stable across bursts. At higher frequencies, however, the response pattern was consistent with frequency-dependent depletion. At 20 Hz, release declined during the second burst but recovered by the third, yielding a U-shaped response profile. At 40 Hz, release dropped to 63.9% of the first burst during the second burst and remained at 62.7% during the third, indicating a sustained reduction without recovery. Together, these findings indicate that high-frequency stimulation places a greater demand on dopaminergic terminals, yet produces only modest depletion of releasable dopamine, suggesting that dopamine pools are rapidly replenished^75^.

In aged animals, the frequency-dependent modulation of release during consecutive bursts was absent (**Extended Fig. 6a,c,e,g**). Overall, dopamine release was reduced, particularly at high frequencies, but remained stable across bursts. Comparisons across age groups showed that aged mice differed significantly from young mice in repeated-burst responses at 40 Hz (Fig. 6g), but not at 5, 10, or 20 Hz (**Extended Fig. 6b,d,f**). At 40 Hz, aged mice showed a reduced response relative to young mice during the first burst only, and this lower level was maintained across subsequent bursts without further decline. Responses during the second and third bursts did not differ from those of young mice. Analysis of response kinetics during the first burst revealed no age effect on rise time (Fig. 6h), but aged mice showed a significantly faster decay (reduced τ, Fig. 6i).

In summary, aged mice showed a frequency-dependent reduction in evoked dopamine release that emerged at higher stimulation frequencies, consistent with impaired dopamine synthesis. In young mice, repeated bursts produce facilitation at low frequencies and modest depletion at high frequencies. This dynamic modulation was absent in aged mice. The deficit was most evident during the first burst at 40 Hz, indicating that aging preferentially impairs the initial high-demand release response rather than the ability to sustain release across repeated bursts. Faster decay kinetics in aged mice further suggest altered dopamine release dynamics.

Together, these findings indicate that aging compresses the dynamic range between tonic- and phasic-like dopaminergic signaling in the LEC. Because novel and salient events normally increase phasic firing, this reduction may diminish the fidelity with which these inputs encode novelty.

### Rescue of Age-related Deficits in Novelty Discrimination

**Figure 7 |:**
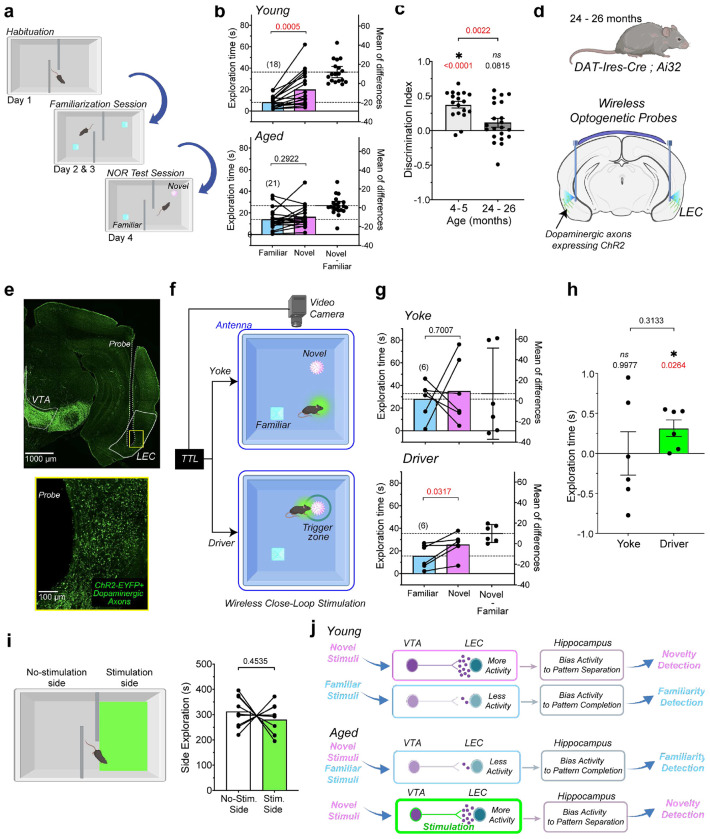
Closed-loop stimulation of dopamine axon terminals in the lateral entorhinal cortex restores novelty discrimination in aged mice. **a**, Schematic of the novel object recognition (NOR) paradigm. **b**, During the test phase, young DAT-IRES-Cre mice preferentially explored the novel object (paired two-tailed t-test, *t*(17) = 4.259), whereas aged DAT-IRES-Cre mice did not (*t*(20) = 1.082). **c**, Discrimination index (DI) was reduced in aged mice relative to young mice (unpaired t-test, *t*(37) = 3.283). Relative to chance (DI = 0), DI differ in young (one-sample t-test, t(17) = 7.986), but did not differ in aged mice (t(20) = 1.834). **d**, Schematic of aged double-mutant mice (DAT-IRES-Cre; Ai32) and wireless optogenetic implants with bilaterally positioned LEDs for stimulation of ChR2-expressing dopaminergic axons. **e**, Representative image of probe placement in the LEC and ChR2 expression in VTA dopamine neurons (*top*) and dopaminergic axons adjacent to the probe tip in the LEC (*bottom*). **f**, Schematic of the closed-loop stimulation paradigm during the NOR test session. The driver animal received stimulation upon entering the novel-object exploration zone; the yoked animal received matched stimulation independent of its own behavior. **g,** Yoked animals did not preferentially explore the novel object (paired two-tailed t-test, *t*(5) = 0.4072; small effect size, Hedges’ *g*_av = 0.27), whereas driver animals did (*t*(5) = 2.954; large effect size, Hedges’ *g*_av = 0.89). **h**, Relative to chance (DI = 0), DI did not differ in yoked animals (one-sample t-test, t(5) = 0.0029), but was significantly above chance in driver animals (t(5) = 3.100). A one-way ANOVA detected no overall group difference (*F*(2,13) = 0.7063). The yoked group showed greater variability than the driver group (SD: 0.665 vs 0.250; variance: 0.442 vs 0.063), with borderline variance difference by F test (F(5,5) = 7.06, *p* = 0.051). **i**, In the RTPP paradigm, stimulation did not induce place preference (paired two-tailed t-test, *t*(8) = 0.7878). **j**, Model proposing the contribution of VTA dopamine-glutamate neurons to novelty and familiarity processing in the LEC-hippocampal circuit in young and aged mice. Data as mean ± SEM. Exact *p* values above the graphs, and sample sizes are shown in parentheses for each group.

Dysfunction of the LEC in older individuals and aged mice has been linked to deficits in novelty discrimination^27, 78^. Dopamine neurons projecting to the LEC respond to novelty with high-frequency burst firing^54^. Because dopamine release in the LEC is reduced, but not abolished, in aged mice, we tested whether stimulation of these dopaminergic terminals during novelty exposure could rescue age-related impairments in novelty discrimination in a novel object recognition (NOR) task. We therefore first confirmed that aged DAT-IRES-Cre mice show impaired performance in this task, which consists of three phases (Fig. 7a): habituation to the open-field arena, a familiarization session in which two identical objects are presented, and a test session in which one familiar object is replaced with a novel object. During the test phase, young DAT-IRES-Cre mice preferentially explored the novel object over the familiar object, indicating intact novelty discrimination (Fig. 7b, **top**). By contrast, aged DAT-IRES-Cre mice did not show a significant difference in exploration time between the novel and familiar objects (Fig. 7b, **bottom**). To quantify this effect across groups, we calculated a discrimination index reflecting relative preference for the novel object. Aged mice showed a significantly lower discrimination index than young mice. One-sample t-tests also showed that discrimination index values were significantly higher than chance performance (0) in young mice, but not in aged mice.

It has been hypothesized that age-related deficits in the NOR task arise from impaired pattern separation, causing animals to treat the novel object as familiar^10^. If so, selectively activating dopamine inputs during exploration of the novel object may be sufficient to rescue performance. To test this hypothesis, we implanted wireless optogenetic probes in aged (24-26 months old) double-mutant mice expressing channelrhodopsin in dopaminergic neurons (DAT-Ires-Cre ; Ai32), with probes positioned to bilaterally stimulate dopaminergic axons in the LEC (Fig. 7d,e; **Extended Fig. 7a**). Using a closed-loop stimulation protocol, photostimulation was delivered only during exploration of the novel object. In this configuration, video tracking detected when the animal’s nose entered a 6 cm zone surrounding the object and triggered a TTL pulse to initiate stimulation (Fig. 7f), which continued until the end of the exploration bout.

If dopamine release must coincide with object investigation to influence novelty processing, then stimulation delivered independently of exploration should fail to rescue the behavioral deficit. To test this prediction, we also implemented a yoked stimulation paradigm that dissociated the amount of stimulation from its behavioral timing (Fig. 7f). Two animals performed the NOR task simultaneously in separate arenas. The driver animal received closed-loop optogenetic stimulation during exploration of the novel object, whereas the yoked animal received identical stimulation pulses delivered independently of its own behavior.

Yoked animals did not preferentially explore the novel object (Fig. 7g, **top**). In contrast, driver animals showed significantly greater exploration of the novel object relative to the familiar object (Fig. 7g, **bottom**). Comparison of discrimination index values did not detect a significant overall difference among groups, mainly due to the large variability of responses observed in the yoke mice (Fig. 7h). However, one-sample t-tests against chance performance (0) revealed that driver animals exhibited significant novelty discrimination, whereas yoked animals did not.

Photostimulation did not alter object exploration time or locomotor activity in driver and yoked mice (**Extended Fig. 7b,c**). Aged mice in which the implant failed to function, which can serve as a no-stimulation control group, also showed deficits in novelty discrimination, with discrimination index values that did not differ significantly from 0 (**Extended Fig. 7d,e**). Together, these findings indicate that stimulation of dopamine axon terminals in the LEC restores novelty discrimination in aged mice only when stimulation is temporally coupled to the animal’s novel exploration behavior.

If stimulation of dopaminergic terminals in the LEC produces an intrinsic reinforcing signal, animals could preferentially occupy locations associated with stimulation and explaining why we observed an increase in novel object exploration. To test this possibility, we performed a real-time place preference (RTPP). In this paradigm, mice freely explored an open-field arena divided into two sides. Entry into the stimulation-paired side triggered optogenetic activation of dopaminergic terminals in the LEC, whereas the opposite side remained unpaired and did not trigger stimulation (Fig. 7i, **left**). Aged mice did not show any preference for the stimulated side (Fig. 7i, **right**), indicating that it was neither rewarding nor aversive.

In summary, stimulation of dopaminergic axons in the LEC rescued performance in the NOR task, likely by enhancing the neural processing underlying novelty detection and pattern separation in the LEC-hippocampus circuit (Fig. 7j) rather than by inducing a simple preference for stimulation-associated locations.

## Discussion

This study identifies a previously unrecognized age-related decline in TH expression, and to a lesser extent VGLUT2, within a subpopulation of DA–GLU neurons projecting to the LEC, revealing their selective vulnerability to aging. This reduced dopaminergic synthetic capacity was accompanied by diminished dopamine release at higher frequencies, thereby narrowing the dynamic range between tonic- and phasic-like firing. Reactivation of DA–GLU terminals in the LEC was sufficient to rescue novelty discrimination deficits in aged mice. These findings establish dopaminergic input to the LEC as a key regulator of novelty detection and show that its dysfunction contributes directly to age-related cognitive impairment.

Using intersectional viral strategies^47, 50, 56^, we selectively labeled DA–GLU and DA-only neurons and found that only INTRSECT-driven EYFP labeling of DA–GLU neurons in the VTA was markedly reduced in middle-aged and aged mice. Two-dimensional reconstruction of DA–GLU neuron distribution across the VTA showed that the reduction was not uniform but instead spared a small hotspot. Given that VTA dopamine neurons are organized into distinct subpopulations that innervate different downstream targets^32–38^, these findings suggest that aging affects some DA–GLU pathways more than others. DA–GLU neurons project densely and selectively to two major targets: the LEC, where we showed that 93% of dopaminergic axons originate from DA–GLU neurons, and the nucleus accumbens shell, whose medial dorsal subregion receives exclusive DA–GLU projections^42^. Here, we found that DA–GLU projections to the LEC do not degenerate, consistent with previous reports^55, 79^, and instead exhibit a pronounced age-related reduction in TH expression. By contrast, a recent study reported no reduction in TH protein in the nucleus accumbens shell or other striatal regions^55^, despite observing decreased TH transcript in VTA dopamine neurons. Those authors found evidence that TH protein levels may be maintained through increased ribosomal translation. These observations support a testable model of target-specific sensitivity to aging, in which DA–GLU projections to the nucleus accumbens shell may preserve TH protein through compensatory mechanisms despite transcript loss, whereas projections to the LEC may be less able to engage such compensation and are therefore more vulnerable. Furthermore, middle-aged mice already showed an approximately 75% reduction in labeled axons in the LEC, consistent with an early onset of this vulnerability.

Our fiber photometry data indicate that the age-related reduction in TH expression has functional consequences, compromising dopamine release in a frequency-dependent manner. The largest deficits emerged during high-frequency stimulation (20 and 40 Hz), consistent with the idea that reduced dopamine synthesis preferentially limits release under conditions of high demand. Dopamine neurons typically fire in a pacemaker-like mode at approximately 4 Hz but transiently shift to burst firing at 20–100 Hz in response to salient environmental events, including novel stimuli and predictive cues^67–69^. Recordings from VTA DA–GLU neurons further show that burst firing in this subpopulation encodes stimulus salience independently of valence, whether rewarding or aversive^80^. In aged mice, the difference in dopamine release between tonic– and phasic–like stimulation in the LEC was blunted, effectively compressing the dynamic range of dopaminergic output. Because phasic dopamine signaling is thought to convey novelty and salience, these findings suggest that this function is particularly vulnerable to age-related reductions in TH expression. At the same time, dopamine release was not abolished in aged mice, indicating that sufficient dopaminergic function remains in the LEC to support signaling. This residual release may reflect compensatory mechanisms that preserve transmission despite reduced TH expression and, importantly, may explain why reactivation of these terminals was still able to rescue novelty discrimination in aged mice.

Compensatory mechanisms could include increased TH enzymatic activity^81^ or greater reliance on vesicle replenishment through DAT-dependent dopamine reuptake. Consistent with the latter possibility, labeling of dopamine neurons using a virus expressing a fluorescent protein under the control of the DAT promoter was unaffected by age. Dopamine signals decayed more rapidly in aged mice, suggesting enhanced DAT-mediated reuptake^82^. Because the same GRABDA2h sensor was used in both groups, differences in intrinsic sensor unbinding kinetics are unlikely to explain this effect. Although lower peak release could still influence decay estimates in practice, these data are most consistent with altered dopamine reuptake and support, but do not conclusively demonstrate, enhanced DAT function.

although faster decay could also reflect lower peak release or differences in GRABDA2h unbinding kinetics. This could be framed as one possible mechanism rather than a conclusion. Closed-loop stimulation of dopaminergic terminals during novel object exploration restored novelty discrimination in aged mice. The absence of improvement in the yoked condition further indicates that rescue depends on coupling dopamine release to exploration of the novel object, rather than on a nonspecific, behaviorally uncoupled increase in dopaminergic tone. Together, these findings show that temporally targeted dopaminergic activation can compensate for age-related circuit dysfunction. Age-related deficits in novelty discrimination in older individuals and aged rodents have been linked to impaired pattern separation, which may bias novel stimuli to be processed as familiar^2, 9, 10, 83^. We propose that age-related deficits in dopamine release contribute to this impairment by reducing the contrast in dopaminergic signaling associated with novel versus familiar stimuli in the LEC. In our data, optogenetic stimulation reinstated a difference between these conditions, consistent with the idea that dopamine amplifies the processing of novel inputs in the LEC (Fig.7j).

Dopaminergic innervation of the LEC is enriched in the superficial layers, where dopamine can modulate layer II neurons through D1-like receptors and facilitate multisensory and cortical excitatory inputs into fan cells^54, 84, 85^. Fan cells in layer IIa of the LEC constitute a major hippocampal-projecting population and send projections to the dentate gyrus^26^, a circuit long implicated in pattern separation^12^. Because DA–GLU inputs to the LEC can provide both dopaminergic modulation and fast excitatory drive through glutamate co-release^53^, they are well positioned to enhance the recruitment of the LEC–dentate gyrus pathway during encounters with novel stimuli. In this framework, loss of dopaminergic signaling in aging would be expected to weaken entorhinal engagement of dentate gyrus circuits, thereby biasing hippocampal processing away from pattern separation and toward CA3-dependent pattern completion (Fig.7j), a functional imbalance proposed to contribute to age-related memory impairment^83^. More broadly, these findings raise the possibility that dopamine in the LEC helps set the threshold for novelty-related information entering the hippocampus, thereby promoting the encoding of distinct representations when new experiences must be distinguished from familiar ones. With aging, this threshold may increase.

Alzheimer’s disease patients and transgenic mouse models show dopaminergic neurodegeneration in the VTA^86, 87^, particularly within its middle portion where DA–GLU neurons are concentrated^86^. Thus, the molecular alterations we identify in DA–GLU neurons projecting to the LEC during normal aging may represent an early vulnerability state that precedes the overt neurodegenerative phenotypes observed in Alzheimer’s disease. In post-mortem tissue from individuals with Parkinson’s disease, there is evidence that melanized dopamine neurons in the substantia nigra lose TH expression before undergoing neurodegeneration^88^. Recent evidence suggests that functional impairments in LEC-projecting dopamine neurons also occur in Alzheimer’s mouse models^89^. This study shows that dopamine-related activity in the LEC was reduced during an associative learning task, with blunted responses to novel odors that produced associative memory deficits. Because these effects were observed in young AD mice, when dopaminergic neurodegeneration is limited, the deficits likely reflect, at least in part, impaired recruitment of LEC-projecting dopamine neurons by salient environmental cues. The rescue with L-DOPA further suggests that reduced dopamine synthesis contributes to this phenotype, paralleling the dopamine-related functional impairments we observe in aged mice. Taken together, these findings position DA–GLU projections to the LEC as an early “weak link” in the mesocorticolimbic system, one that could cascade into a disproportionate disruption of LEC-hippocampal-dependent memory and salience processing.

## Methods

### Experimental Animals

All procedures were conducted in accordance with National Institutes of Health *Guide for the Care and Use of Laboratory Animals* and approved by the Institutional Animal Care and Use Committee of the City University of New York (CUNY) Advanced Science Research Center (ASRC). Mice were housed under standard barrier conditions with a reverse 12-hour light/dark cycle and ad libitum access to food and water. Male and female mice were used for all experiments. Three transgenic lines were utilized: (1) TH-Flp;VGLUT2-Cre mice for INTRSECT viral experiments and (2) DAT-IRES-Cre mice for behavioral testing and ChR2-YFP viral labeling (3) DAT-IRES-Cre/+ ; Ai32 mice expressing ChR2-YFP in all DAT-expressing dopaminergic neurons. Animals were aged to 2-3 months (young), 13-14 months (middle), or 23-24 months (old).

### Stereotactic Surgery

#### INTRSECT Viral Strategy.

To selectively label DA–GLU and DA-only VTA neurons, TH-Flp;VGLUT2-Cre mice were injected with a mixture of AAV8-EF1a-Con/Fon-EYFP-WPRE and AAV8-EF1a -Coff/Fon-mCherry-WPRE^56^. The viruses were kindly donated by Dr. Deisseroth’s lab. Injections were performed at 2, 13, or 23 months of age to allow one month of viral expression prior to tissue collection at 3, 14, or 24 months (n = 9, 7, and 5, respectively). Mice were anesthetized with isoflurane (3% induction, 2% maintenance), and 1 μL of viral solution (1.5 × 10^12^ vg/mL) was pressure-injected into the unilateral medial VTA and lateral VTA/SNc. Stereotaxic coordinates (relative to bregma) were adjusted by bodyweight: medial VTA: AP −3.0 to −3.4 mm, ML ±0.5 mm, DV −4.1 to −4.5 mm; lateral VTA/SNc: AP −3.0 to −3.4 mm, ML −1.3 mm, DV −4.3 mm. The pipette remained in place for 5 minutes to minimize backflow.

#### ChR2-YFP Strategy.

To label DAergic axons independent of TH or VGLUT2 promoter activity, DAT-IRES-Cre mice were injected bilaterally in the VTA with AAV5-EF1a-DIO-ChR2(H134R)-EYFP (UNC Lot # AV4313-2B; 1.5 × 10^12^ vg/mL) at either 2 months (n = 10) or 23 months (n = 11). Coordinates for the medial VTA and procedure matched the INTRSECT protocol above.

#### Fiber Optic Implantation Combined with Viral Injections.

To express a red-shifted opsin, DAT-IRES-Cre mice received unilateral injections of AAV8-nEF1-Con/Foff 2.0-ChRmine-oScarlet (Addgene #137161; 2.16 x 10^12^ vg/mL) into the VTA using the medial VTA coordinates described above. Mice also received bilateral injections of AAV9-hsyn-GRAB_DA2h (Addgene #140554; 1.88 x 10^12^ vg/mL) into the LEC (AP −3.4 mm, ML −0.5 mm, DV −3.8 to −3.9 mm, angle 8°). A fiber optic cannula (black ceramic ferrule, 1.25 mm ferrule diameter; 200 μm core; NA = 0.37; length = 5.0 mm; Neurophotometrics) was implanted into the LEC at the same coordinates used for the GRAB_DA2h_ injection.

#### Implantation of Wireless Optogenetic Probes.

After induction and maintenance of surgical anesthesia with isoflurane, the skull was exposed, and target coordinates were determined relative to bregma, LEC: −3.5 mm anteroposterior; ±4.1 mm mediolateral; −4.2 mm (males) and −4.0 ( females) dorsoventral; 0° angle). Implantable Devices (5 mm; NeuroLux, Northfield, IL, USA) were prepared by attaching each probe to a Custom Stereotactic Implant Adapter Surgical Clip (NeuroLux, Northfield, IL, USA) using an alligator-style connector tooth. The Surgical Clip was then secured into the stereotaxic adaptor, and the probe was positioned above the burr hole and lowered slowly into the brain. Following successful placement, the implant was secured with Loctite 454 followed by accelerator to quickly harden it, and the incision was closed using Vetbond Tissue Adhesive (3M Center, St. Paul, MN, USA). Animals were monitored during the immediate postoperative period for signs of distress or pain and received postoperative monitoring and supportive care until full recovery. Surgeries were performed at least two weeks prior to experimental testing to allow adequate recovery before data acquisition and tissue collection.

### Immunohistochemistry

#### INTRSECT-Labeled Brains.

Immunohistochemistry (IHC) was performed as previously described^48^. Mice were anesthetized (ketamine/xylazine) and perfused with PBS followed by 4% paraformaldehyde (PFA). Brains were post-fixed and cut into 50 μm coronal sections in a vibratome (MicroSlicer DTK-1000N). Sections underwent sequential washes in 1x PBS (3 x 10 minutes), followed by incubation in 0.1 M glycine, washed in 1x PBS again (3 x 10 minutes), then were blocked in 10% normal goat serum (NGS) with 0.1% Triton X-100 and incubated with primary antibodies: anti-mCherry (rat, 1:5000; Thermo Fisher Scientific Cat# M11217, RRID:AB_2536611), anti-GFP (chicken, 1:5000; Thermo Fisher Scientific Cat# A10262, RRID:AB_2534023) and either anti-TH (rabbit, 1:5000; Thermo Fisher Scientific Cat# OPA1-04050, RRID:AB_325653) or anti-VGLUT2 (rabbit, 1:250–1:5000; Synaptic Systems #135403; RRID:AB_887883). Secondary antibodies anti-rat Alexa Fluor 568 (goat, 1:200; Thermo Fisher Scientific Cat# A-11077, RRID:AB_2534121) anti-chicken Alexa Fluor 488 (goat, 1:200; Thermo Fisher Scientific Cat# A32931, RRID:AB_2762843) and anti-rabbit Alexa Fluor 647 (goat, 1:200 to 1:400; Thermo Fisher Scientific Cat# A32733, RRID:AB_2633282) diluted in 0.02% PBS-T.

#### ChR2-YFP Labeled Brains.

Sections were processed similarly as the INTRSECT-labeled brains but blocked with an adapted aging-specific solution (Schnell, Staines et al. 1999) prepared using PGBA, 10% NGS, and 0.5% Triton X-100. Primary antibodies for TH quantification: anti-GFP (rabbit, 1:5000; Abcam Cat# ab6556, RRID:AB_305564) and anti-TH (chicken, 1:5000; Aves Labs Cat# TYH, RRID:AB_10013440). Primary antibodies for VGLUT2 quantification: anti-GFP (chicken, 1:5000; Thermo Fisher Scientific Cat# A10262, RRID:AB_2534023) and anti-VGLUT2 (rabbit, 1:1000: Synaptic Systems Cat# 135403, RRID:AB_887883). Secondary antibodies for TH quantification: anti-rabbit Alexa Fluor Plus 647 (goat, 1:400; Thermo Fisher Scientific Cat# A32733, RRID:AB_2633282) and anti-chicken Alexa Fluor 568 (goat, 1:400; Thermo Fisher Scientific Cat# A-1104, RRID:AB_2534098). Secondary antibodies for VGLUT2 quantification: anti-chicken Alexa Fluor Plus 647 (goat, 1:400; Thermo Fisher Scientific Cat# A32933, RRID:AB_2762845) and anti-rabbit Alexa Fluor 532 (goat, 1:400; Thermo Fisher Scientific Cat# A-11009, RRID:AB_2534076). Secondary antibodies then underwent a wash in deionized water (5 minutes). Lipofuscin autofluorescence was quenched using cupric sulfate (CuSO) in ammonium acetate (50 mM, pH 5.0; 15 minutes) following Schnell et al., 1999. A subsequent wash in deionized water (5 minutes) followed, after which sections underwent additional washes in 1x PBS (3 x 10 minutes) and mounted.

### In Situ Hybridization (RNAscope)

*In situ* hybridization was combined with immunohistochemistry (IHC) using RNAscope reagents (Advanced Cell Diagnostics; ACD Bio) as previously described^46^ and was performed by the Epigenetics Core Facility at the CUNY Advanced Science Research Center (ASRC). Tissue sections were fixed in chilled 4% paraformaldehyde for 15 min at 4 °C, dehydrated through graded ethanol, and air-dried for 5 min. Endogenous peroxidase activity was quenched with hydrogen peroxide for 10 min, followed by antigen retrieval for 5 min in boiling buffer. Sections were then processed for IHC with anti-GFP (chicken, 1:1000) and anti-mCherry (rat, 1:1000), followed by protease digestion for 30 min at 40 °C. For RNAscope, the vesicular glutamate transporter 2 probe (Slc17a6/VGLUT2; ACD Bio, #1170921-C2) was hybridized for 2 h at 40 °C in a humidity-controlled oven (HybEZ II, ACD Bio). Signal amplification was performed using RNAscope AMP reagents (ACD Bio), and probe signal was visualized using probe-specific horseradish peroxidase–based detection with Opal 690 dye (PerkinElmer; 1:1500). Slides were subsequently incubated with secondary antibodies (anti-chicken Alexa Fluor 488, 1:400; anti-rat Alexa Fluor 568, 1:400), counterstained with DAPI, and coverslipped with ProLong Gold Antifade mounting medium (Thermo Fisher Scientific).

### Image Acquisition and Quantification

#### INTRSECT quantification in the VTA.

Some images were acquired on a Leica DM6 B epifluorescence microscope, and others on a Zeiss LSM 880 confocal microscope. Because quantification did not differ across imaging modalities, data from both systems were pooled for analysis. Neuronal subtypes within the VTA were quantified in ImageJ. For each z-stack, a z-projection was generated to create a reference image for counting. Raw optical sections were viewed in parallel to confirm signal localization within somata and to avoid misclassification due to projection artifacts. Manual cell counts were performed using the ImageJ Multi-point Tool. Each neuronal phenotype (and each of the seven combinatorial marker categories) was assigned a unique counter index: TH-only, mCherry-only, EYFP-only, TH+mCherry, TH+EYFP, TH+mCherry+EYFP, and mCherry+EYFP. Cells were classified using predefined inclusion criteria based on marker colocalization and somatic morphology. Each counted cell was marked on the z-projected image and then verified against the corresponding raw z-stack to ensure accurate attribution of fluorescence within individual cell bodies. Cell counts and x–y coordinates were exported from ImageJ and compiled in Microsoft Excel for organization and downstream analysis. Total counts per subtype and per section were calculated from the exported files prior to statistical analysis. The exported x–y coordinates were used to generate 2D spatial plots of cell distributions.

#### INTRSECT and VGLUT2 RNAscope Quantification in the VTA.

Images were acquired on a Zeiss LSM 880 confocal microscope. All analyses were performed in Imaris (Bitplane/Oxford). The VTA was defined in Imaris by manual surface delineation. Boundaries were traced on the first optical section and propagated across the full z-stack. The VTA surface was then used to mask the relevant fluorescence channels to restrict analysis to the region of interest (ROI) (mCherry and EYFP channels). Cell bodies were segmented from the masked neuronal fluorescence channels as 3D surfaces using Imaris surface creation (smoothing enabled; surface detail = 2.0 μm), with intensity thresholding and background subtraction applied to separate signal from background. Segmented neuronal surfaces were manually curated to remove artifacts, merge fragmented objects, and split incorrectly merged cells. VGLUT2 mRNA transcripts were quantified using Imaris spot detection on the masked VGLUT2 transcript channel. The spot (XY) diameter was determined empirically by measuring puncta in slice view and then held constant across animals within the VTA. To quantify VGLUT2 puncta per neuron, the Imaris Cell to Vesicle function was used. Pre-segmented neuronal surfaces (mCherry+ and EYFP+ cells) were imported as “cell” objects, and detected VGLUT2 spots were imported as “vesicle” objects. Spots were classified as intracellular for a given neuron when the distance from the spot to that neuron’s surface was zero; spots with nonzero distance were considered extracellular relative to that neuron. Per-cell transcript abundance was extracted as “Cell number of vesicles,” representing the number of VGLUT2 spots assigned to each neuronal surface.

#### INTRSECT and ChR2-EYFP Axonal Quantification in the LEC.

Images were acquired on a Leica DM6 B epifluorescence microscope. All analyses were performed in Imaris (Bitplane/Oxford). In Imaris, the LEC ROI was manually delineated using anatomical boundaries and masks were generated on the relevant fluorescence channels (EYFP and/or mCherry) to restrict subsequent analysis to the selected signal. Within the selected LEC ROI, labeled axons were segmented using Imaris surface reconstruction without smoothing or background subtraction. Total axonal volume for each region was obtained from the Imaris surface statistics and used as the primary measure of axonal signal within the LEC and normalized by the total volume of the LEC.

#### **TH Inside ChR2-EYFP+ Axons Quantification in the LEC**.

Images were acquired on a Leica DM6 B epifluorescence microscope and analyzed in Imaris (Bitplane/Oxford). For each section, regions of interest corresponding to superficial and deep LEC layers were defined using anatomical landmarks. The analysis window was held constant for each ROI (x–y dimensions fixed at 1000 × 1000 pixels, with a z-stack of 15 optical planes). Surface segmentation was restricted to the appropriate ROI to generate superficial and deep EYFP surfaces (EYFP+ axons) and superficial and deep TH surfaces (TH signal in axons). Colocalization was assessed by filtering TH surfaces based on their overlap with the corresponding EYFP surface using an overlapped volume ratio metric; TH signal was classified as within EYFP+ axons when the overlap ratio was ≥0.65. For each superficial and deep LEC ROI, the total TH volume classified as inside versus outside EYFP+ axons was extracted and recorded.

#### VGLUT2 Inside ChR2-EYFP+ Axons Quantification in the LEC.

Images were acquired on a Zeiss LSM 880 confocal microscope equipped with Airyscan. Quantification followed the same analysis pipeline described for “TH Inside ChR2-EYFP+ Axon Quantification in the LEC”. Surfaces representing EYFP+ axons and VGLUT2+ axonal terminals were generated and filtered using the same overlap criteria of ≥0.65. For each superficial and deep LEC ROI, the total volume of VGLUT2 signal classified as within EYFP+ axons was extracted and recorded.

### Dual Color Fiber Photometry with Optogenetic Stimulation

#### ChRmine Stimulation and GRAB_DA2h_ Recordings.

Fiber photometry recordings were acquired using a Neurophotometrics FP3002 system. Optogenetic stimulation was delivered through the LEC recording fiber optic cannula using a 635 nm laser (3 mW at the patch-cord tip) at 5, 10, 20, and 40 Hz (5 ms pulse width). For each frequency, stimulation consisted of a 60-s burst delivered three times with an inter-trial interval (ITI) of 120 s. The 5-, 10-, and 20 Hz conditions were presented in randomized order and separated by 30 minutes; the 40 Hz condition was always delivered last to avoid potential carryover effects of high-frequency stimulation on responses measured at lower frequencies. Simultaneously, GRAB_DA2h_ fluorescence was excited with a 470 nm LED (20 μW at the patch-cord tip). Emitted fluorescence was collected through a green bandpass filter and sampled at 30 Hz.

Analysis of the dopamine sensor signal was performed using custom-written Python scripts (MetaCell). The 470 nm channel was detrended to correct photobleaching by fitting a best-fit line across the entire session and subtracting it from the raw signal, yielding ΔF/F. Signals were smoothed using a 10 s rolling window, and peri-event time histograms were generated on a trial-by-trial basis from −2 to +180 s relative to stimulation onset. Baseline was defined as the mean signal from −2 to 0 s, and responses were expressed as Z-scored, baseline-normalized ΔF/F (normalized ΔF). To quantify stimulation-evoked dopamine release, area under the curve (AUC) was calculated from 0–120 s relative to stimulation onset. For decay kinetics (τ_decay), the maximum baseline-corrected response was identified within a post-onset peak-search window of 0–65 s to obtain peak amplitude and its time (t_peak; used as the rise-time parameter). The decay phase was fit from t_peak to 180 s with a single-exponential model, y(t) = A·exp(–(t – t_peak)/τ_decay), using nonlinear least-squares minimization with constraints A > 0.5 and 0.05 ≤ τ_decay ≤ 500 s. Goodness-of-fit was assessed using the coefficient of determination (R^2^) computed over the fit window. For traces with poor fits (R^2^ < 0.7), an alternative approach was used to prevent the offset term from dominating the fit: the offset was estimated as the mean signal from 95–100 s, and the decay segment (65–100 s) was offset-corrected and fit with a no-offset exponential model.

### Behavioral Experiments with Close-Loop Optogenetics

#### Novel Object Recognition (NOR) Task.

Habituation and experimental sessions were conducted in either (1) custom-built open-field arena constructed from white plastic (60 × 40 × 30 cm), with two dark blue walls (approximately 10 cm high) dividing the arena into two connected compartments of equal size, or (2) blue open-field chambers (40 × 40 × 40 cm; MazeEngineers, USA) used for the closed-loop optostimulation experiment. Before testing, animals underwent three consecutive days of experimenter handling in the colony housing room, lasting 5 to 10 minutes per day. All sessions were conducted during the dark phase of the reversed light cycle, which began at 11:00 AM. Mice were transferred to a room adjacent to the testing room, and allowed to acclimate for a minimum of 15 minutes alone in home cages. On Day 1, mice were habituated to the open field arena without objects for 10 minutes. During the subsequent two-day familiarization phase, mice completed daily 10-minute sessions in which they explored two identical objects positioned opposite each other. On Day 4, a 10-minute NOR test was conducted in which one familiar object was replaced with a novel object. Objects measured 5 cm x 5 cm and were three-dimensionally printed in dark blue at the ASRC. Object designs were based on those previously described^90^. The arena and objects were cleaned with 70% ethanol between trials. Animals were tested individually, except during the closed-loop stimulation experiments, in which mice were tested in pairs in two adjacent arenas.

#### Closed-Loop Wireless Optogenetic Stimulation.

Closed-loop optogenetic stimulation was delivered during the NOR test using a modular NeuroLux platform configured to control two experimental arenas simultaneously and activate wireless optogenetic implants (NeuroLux, IL, USA). The battery-free implants were powered by near-field electromagnetic energy delivered by an external transmitter to a flexible loop antenna on the implant, which was converted into electrical power to drive a microscale LED (μLED) for light delivery to opsin-expressing neurons. Stimulation protocols were programmed using the manufacturer’s graphical user interface. Stimulation was triggered by a TTL pulse from an EthoVision-connected video motoring device and delivered at 20 Hz with 5 ms pulses. EthoVision was configured to trigger TTL output whenever the driver animal’s nose entered a 6 cm zone surrounding the novel object. TTL pulse was simultaneously delivered to the adjacent arena, such that the yoked animal received wireless photostimulation at the same time.

#### Real-Time Place Preference (RTPP) with Wireless Optogenetic Stimulation.

Mice were placed in a custom-built two-compartment conditioning apparatus, as described above, and habituated to it for one day (10 min). On the subsequent mice were tested on a RTPP paradigm (10 min session) in which wireless photostimulation (20 Hz, 5 ms pulse width) was delivered whenever the animal entered and remained in the stimulation-paired compartment, whereas no stimulation was delivered in the opposite compartment. Stimulation side was randomized between animals (half of the animals on the right and half of the left compartment). Wireless stimulation was triggered by a TTL pulse from an EthoVision-connected video monitoring system and was programmed to turn on whenever the animal’s center point was detected within a zone corresponding to the stimulation-paired compartment.

### Statistical Analyses

All statistical tests are reported in the figure legends. Statistical analyses were performed using GraphPad Prism or RStudio; with α set at 0.05. Data distributions were assessed using the Shapiro–Wilk test to determine whether parametric or nonparametric tests were appropriate. Between-group comparisons were analyzed using one-way ANOVA, two-way ANOVA, or repeated-measures ANOVA, followed by Dunnett’s or Bonferroni post hoc tests. For the frequency effect of GRAB_DA2h_, and based on an a priori directional hypothesis that age-related reductions would be most pronounced at higher stimulation frequencies, we performed planned comparisons at 20 Hz and 40 Hz using one-tailed unpaired Welch’s t-tests (Young > Aged), with Holm–Bonferroni correction across the two tests. For non-normally distributed data, Kruskal– Wallis and Mann–Whitney U tests were used with Dunn’s post hoc correction when appropriate. Effect sizes are reported as partial eta squared (ηp^2^), Cohen’s d, or rank-biserial r, depending on the analysis.

To resolve the spatial distribution of neurons in the VTA, we labeled two points in each image, for each age category, that are indicative of the mid-line. Using these points, a straight line was drawn, and the distance of each cell type of interest from this midline was estimated. This is reflective of the medial-lateral distribution of the neurons. Owing to variation in size of the brain sections, we normalized the distances from the midline for each image to the longest distance among all TH+ neurons. This enabled us to spatially compare the distributions of DA-only (mCherry+), DA–GLU (EFYP+), and TH+ neurons within, and between ages. For each image and each neuronal group, the normalized distances were used to compute a histogram. This allowed us to count the number of neurons at specific distances from the midline. For each age group, all this information was then sorted into a two-dimensional spatial map for each replicate animal, with bregma along one axis, medial-lateral distance on the other axis, and the counts of cells of interest for each combination of bregma and medial-lateral distance on the color axis. The spatial maps were then averaged over replicate animals for each age group, and the averaged spatial maps are represented as heatmaps. Difference heatmaps are generated by subtracting the averaged spatial maps. To compare the differences between spatial maps of different age groups, we carried out multiple one-sampled t-tests. For each bregma and medial-lateral distance, the replicate counts for 14- and 24-month-old animals were tested for statistical significance using a one-tailed t-test against the mean of the 3-month-old group. Owing to the large number of t-tests, we then corrected the p-values using a Benjamini-Hochberg correction to adjust the FDR to 5%. Only the corrected p-values<0.05 were considered significant and are represented in the p-value heatmaps. All analyses, plots, and statistical comparisons were carried out using custom R scripts.

## Supplementary Material

1

This is a list of supplementary files associated with this preprint. Click to download.

SupplementaryVideo1YoungMouse.mov

SupplementaryVideo2AgedMouse.mov
