# Supplementary material for: Aging-related vulnerability in dopamine–glutamate neurons weakens entorhinal dopamine signaling and underlies novelty discrimination deficits
Source: Res Sq. 2026 Apr 26:rs.3.rs-9321992. Preprint. [Version 1] doi: 10.21203/rs.3.rs-9321992/v1 (PMC13131891; doi:10.21203/rs.3.rs-9321992/v1)
Supplement: 1 [file NIHPPrs9321992v1-supplement-1.pdf]

Supplementary Information

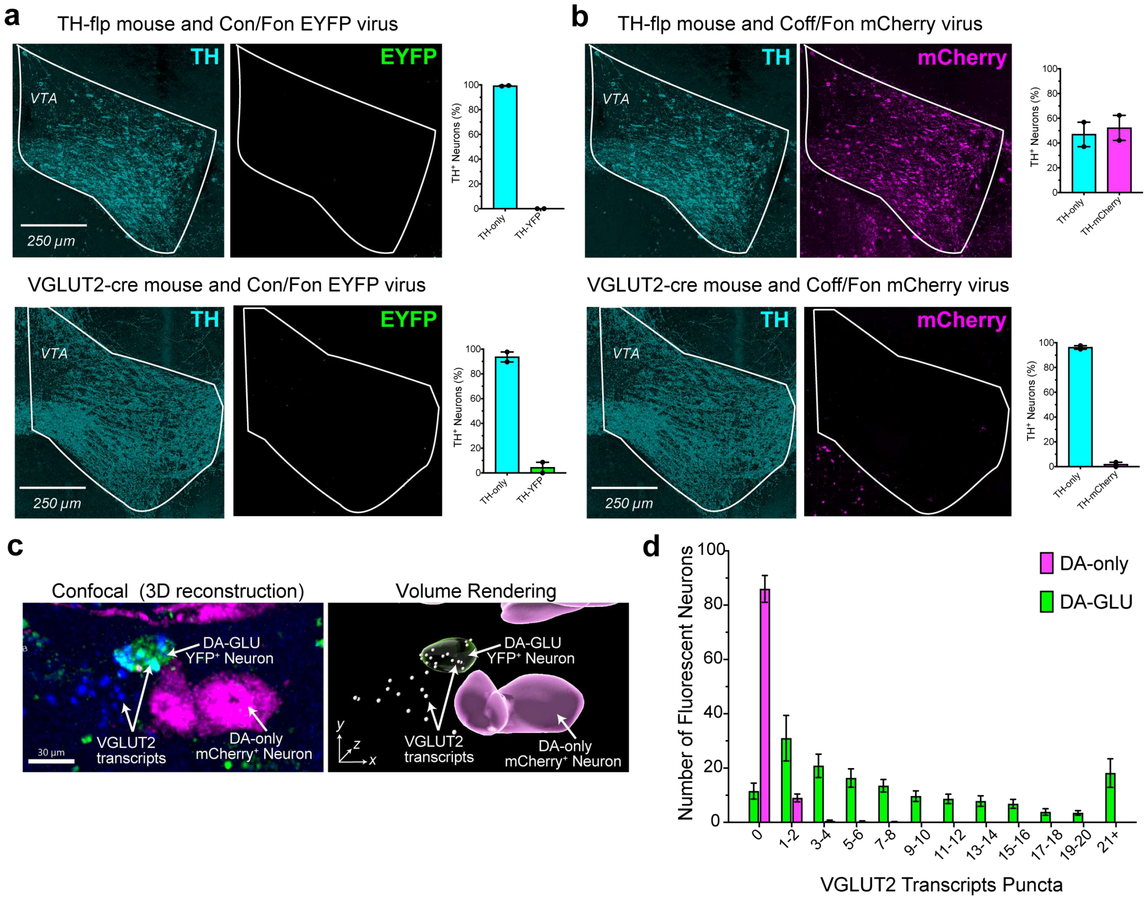

Extended Fig. 1 | Validation of INTRSECT recombinase dependence. **a**, Representative photomicrographs showing TH and EYFP immunoreactivity in TH-Flp (top) and VGLUT2-Cre (bottom) mice injected with an INTRSECT Con/Fon-EYFP virus. Quantification (right) shows no EYFP expression, confirming dual recombinase dependence. **b**, Representative photomicrographs showing TH and mCherry immunoreactivity in TH-Flp (top) and VGLUT2-Cre (bottom) mice injected with an INTRSECT Coff/Fon-mCherry virus. Quantification (right) shows robust mCherry expression in TH-Flp mice but no expression in VGLUT2-Cre mice, consistent with Flp-dependent activation and Cre-dependent suppression. **c**, Confocal images (left) and 3D

renderings (right) showing VGLUT2 (Slc17a6) transcript puncta (blue) within EYFP+ neurons (green; DA–GLU) and mCherry+ neurons (magenta; DA-only). **d**, Quantification of VGLUT2 transcript puncta per neuron in EYFP+ versus mCherry+ populations shows that VGLUT2 (a glutamatergic marker) is enriched in EYFP+ neurons, whereas mCherry+ neurons exhibit little to no VGLUT2 signal, with only a few cells containing a single punctum.

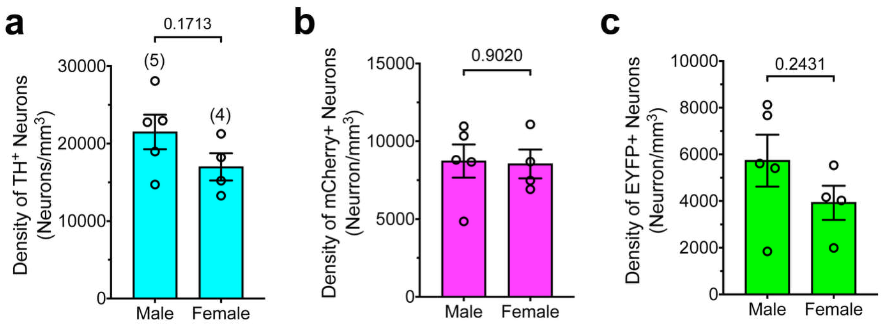

Extended Fig. 2 | No sex differences in dopaminergic subpopulations density in the VTA of young mice. Bar graphs comparing the density (neurons/mm³) of TH+ neurons (**a**), mCherry+ neurons (**b**), and EYFP+ neurons (**c**) in male versus female young mice. Each bar represents group mean ± SEM. P-values are displayed on the plots, indicating no statistically significant differences between sexes for any neuronal population shown.

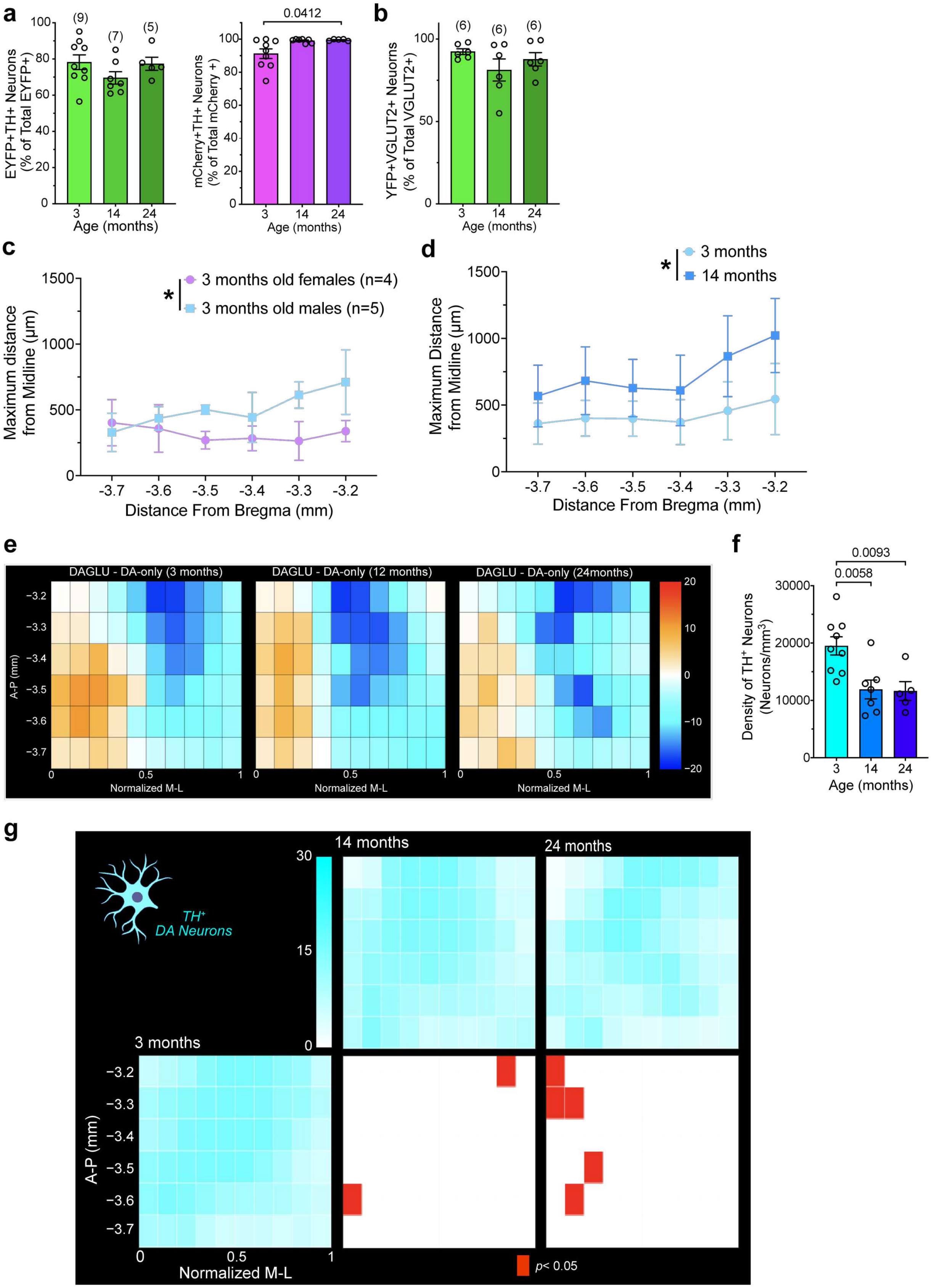
a
EYFP+TH+ Neurons (% of Total EYFP+)
(9)
(7)
(5)
3
14
24
Age (months)
mCherry+TH+ Neurons (% of Total mCherry +)
0.0412
Age (months)
b
YFP+VGLUT2+ Neurons (% of Total VGLUT2+)
(6)
(6)
(6)
Age (months)
c
Maximum distance from Midline (μm)
3 months old females (n=4)
3 months old males (n=5)
Distance From Bregma (mm)
d
Maximum Distance from Midline (μm)
3 months
14 months
Distance From Bregma (mm)
e
DAGLU - DA-only (3 months)
DAGLU - DA-only (12 months)
DAGLU - DA-only (24months)
A-P (mm)
Normalized M-L
f
Density of TH+ Neurons (Neurons/mm3)
0.0093
0.0058
Age (months)
g
TH+ DA Neurons
14 months
24 months
3 months
A-P (mm)
Normalized M-L
p< 0.05

Extended Fig. 3 | Validation of INTRSECT labeling in aged mice and age-related distribution of dopaminergic subpopulations in the VTA. a, Specificity of INTRSECT-labeled neurons for TH+ dopamine neurons at 3, 14, and 24 months. Left, percentage of EYFP+TH+ neurons among all EYFP+ cells; right, percentage of mCherry+TH+ neurons among all mCherry+ cells. Viral specificity was maintained with age, with a modest increase in TH co-localization in the mCherry+ population at 24 months (EYFP: no age effect, one-way ANOVA, $F(2,18)=0.9193$; mCherry: Kruskal–Wallis, $H=6.992$, Dunn's post hoc; significant adjusted p-values indicated). Numbers above bars indicate mice. **b**, Validation of glutamatergic identity of EYFP+ neurons using EYFP immunoreactivity combined with in situ hybridization for VGLUT2 (*Slc17a6*). Plot shows the percentage of EYFP+VGLUT2+ neurons among all VGLUT2+ neurons at 3, 14, and 24 months. Each dot represents a section (4 mice per age, 6 sections per mouse). **e**, 2D VTA density maps showing the difference in spatial distribution between EYFP+ (DA–GLU) and mCherry+ (DA-only) cells across anterior–posterior (A–P) and normalized medial–lateral (M–L) coordinates. Color indicates the DA–GLU – DA-only difference per bin: white denotes no difference, warm colors indicate bins enriched in DA–GLU neurons, and cool colors indicate bins enriched in DA-only neurons. **f**, Bar graph showing TH+ neuron density (neurons/mm^3) across age groups (one-way ANOVA main effect of age: $F_{(2,18)} = 7.91$, $p=0.0034$, large effect size: $\eta p^2=0.47$; Dunnett's post hoc test vs 3 months; exact p values shown above brackets). **g**. 2D VTA density map showing the distribution of TH+ neurons across anterior–posterior (A–P) and normalized medial–lateral (M–L) coordinates. Red tiles indicate bins that differed from the 3-month group (one-tailed t-test vs the 3-month mean), with p-values Benjamini–Hochberg corrected to control the false discovery rate (FDR) at 5%; only adjusted $p < 0.05$ bins are shown.

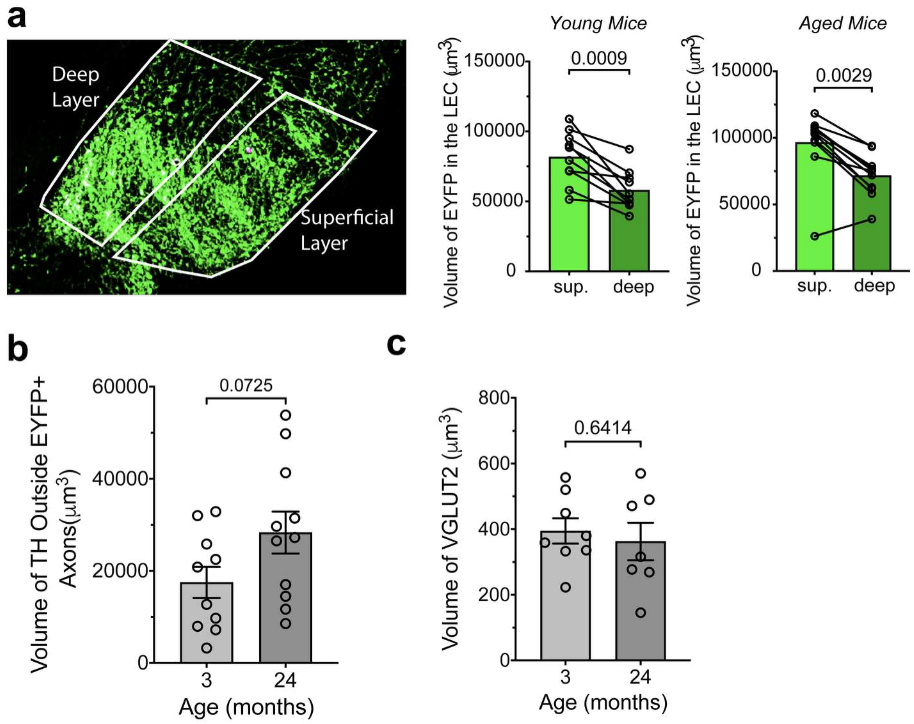

Extended Fig. 4 | Layer- and age-dependent analysis of EYFP, TH, and VGLUT2 volume in the LEC. a, Left, representative LEC image illustrating the delineation of superficial and deep layers for volumetric analysis of ChR2–EYFP labeled axons in DAT-IRES-Cre mice. Bar plots show total EYFP+ axonal volume (μm^3) in superficial versus deep layers at 3 (*left*) and 24 months (*right*). EYFP+ volume was greater in superficial than deep layers at both ages (3 months: paired t-test, $t(9)=4.882$, $P=0.0009$; 24 months: Wilcoxon matched-pairs signed-rank test, $W = -62$, $P=0.0029$). **b**, TH+ axonal volume (μm^3) outside EYFP+ axons in the LEC at 3 and 24 months; no age difference (unpaired t-test, $t(19)=1.876$, $P=0.0761$). **c**, Total VGLUT2+ terminal volume (μm^3) in the LEC at 3 and 24 months; no age difference (unpaired t-test, $t(13)=0.4768$, $P = 0.6414$). **d**, VGLUT2+ volume (μm^3) in superficial and deep layers at 3 and 24 months; no age effect (unpaired t-test, $t(13)=0.4768$, $P = 0.6414$). Each dot represents an animal; bars show mean ± SEM.

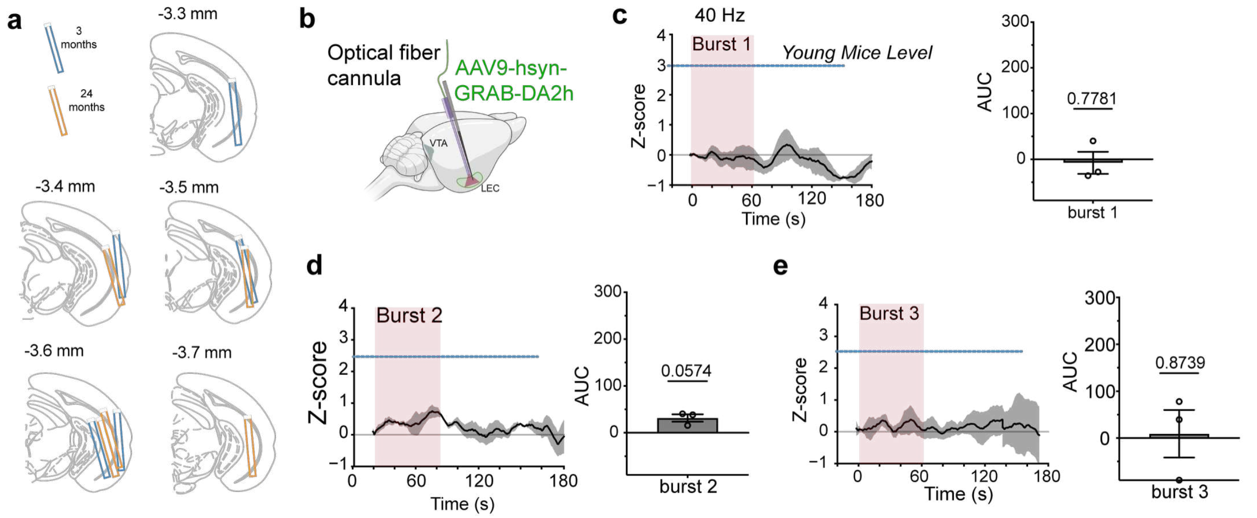

Extended Fig. 5 | Validation of the combined optogenetics and fiber photometry experiment. a, Schematic of optic fiber cannula placement in young and aged mice. **b**, Schematic of injection of $GRAB_{DA2h}$ virus and cannula placement for control experiments testing the effect of red-light stimulation on $GRAB_{DA}$ signals. **c–e**, Peri-event histograms showing mean $GRAB_{DA}$ responses to 40 Hz stimulation delivered as three consecutive 60-s bursts (n = 3 mice; 2 young, 1 aged; shaded area, ± SEM). Right, quantification of red-light effects on $GRAB_{DA}$ signals for bursts 1–3; one-sample t-tests showed no significant effect (burst 1: t(2)=0.3218, P=0.7781; burst 2: t(2)=3.991, P=0.0574; burst 3: t(2)=0.1797, P=0.8739).

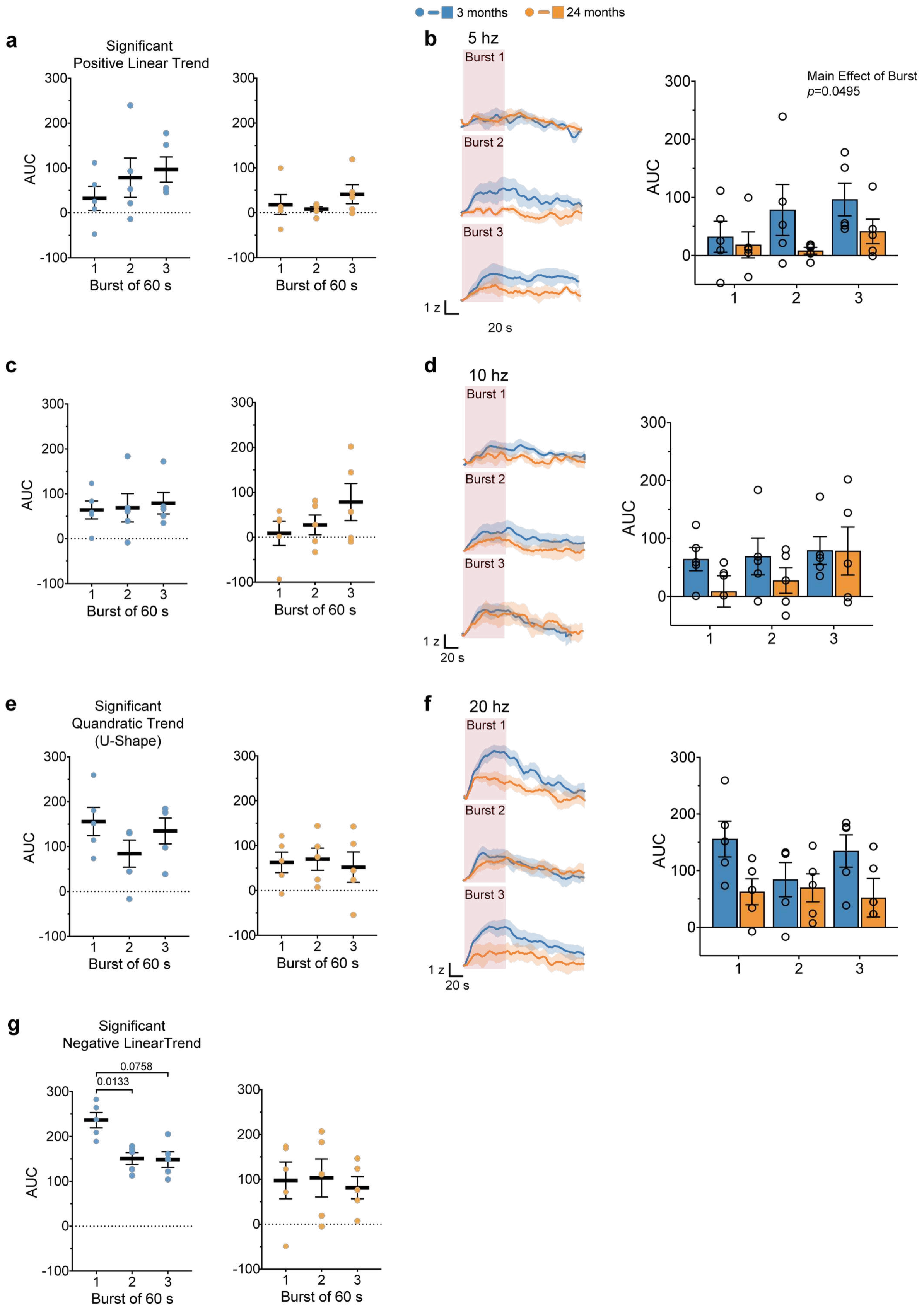

3 months
24 months
a
Significant
Positive Linear Trend
AUC
Burst of 60 s
b
5 hz
Burst 1
Burst 2
Burst 3
1 z
20 s
Main Effect of Burst
p=0.0495
c
d
10 hz
e
Significant
Quandratic Trend
(U-Shape)
f
20 hz
g
Significant
Negative LinearTrend
0.0758
0.0133

Extended Fig. 6 | GRABDA2h measurements of dopamine release in response to three consecutive bursts at different stimulation frequencies in young and aged mice. a,c,e,g, Dopamine release, measured as area under the curve (AUC), across three consecutive 60-s stimulation bursts in young (left, blue) and aged mice (right, orange). Although the omnibus repeated-measures one-way ANOVA was not significant at 5, 10, or 20 Hz, planned trend analyses revealed frequency-dependent changes across bursts in young mice. At 5 Hz (**a, left**), a planned within-subject linear trend analysis showed a significant increase across bursts, consistent with short-term facilitation ($t(4) = 5.09$, $p = 0.007$). At 10 Hz (**c, left**), dopamine release did not change across bursts ($t(4) = 0.56$, $p = 0.605$). At 20 Hz (**e, left**), a planned within-subject quadratic trend analysis revealed a significant U-shaped pattern across bursts ($t(4) = 5.03$, $p = 0.0073$), indicating that the response was lower during the second burst than during the first and third bursts. At 40 Hz (**g, left**), a planned within-subject linear trend analysis revealed a significant decrease across bursts ($t(4) = -2.87$, $p = 0.045$). In young mice at 40 Hz, the repeated-measures one-way ANOVA was also significant; exact p values are shown above the brackets. In aged mice (**a,c,e,g, right**), no significant linear or quadratic trends were detected at any frequency.

b, Mean traces (young, blue; aged, orange; shaded area indicates ± s.e.m.) and quantification for 5 Hz stimulation across three consecutive bursts. Two-way repeated-measures ANOVA showed no main effect of age and no age × burst interaction, but a main effect of burst (age × burst: $F(2,16) = 1.607$, $p = 0.2312$; burst: $F(2,16) = 3.648$, $p = 0.0495$; age: $F(1,8) = 1.935$, $p = 0.2016$). **d**, Same as in b, for 10 Hz stimulation. No significant effects were detected (age × burst: $F(2,16) = 0.6000$, $p = 0.5607$; burst: $F(2,16) = 1.428$, $p = 0.2826$; age: $F(1,8) = 1.432$, $p = 0.2657$). **f**, Same as in b, for 20 Hz stimulation. No significant effects were detected (age × burst: $F(2,16) = 2.824$, $p = 0.0890$; burst: $F(2,16) = 1.620$, $p = 0.2287$; age: $F(1,8) = 3.222$, $p = 0.1104$).

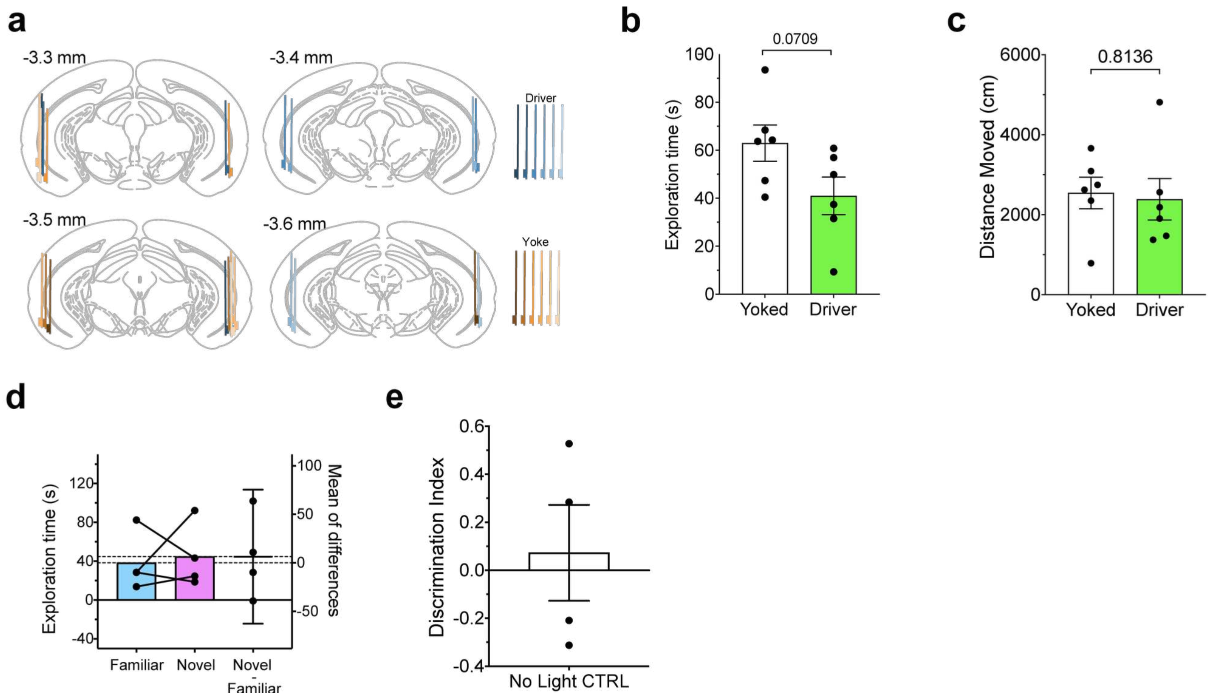

Extended Fig. 7 | Closed-loop optogenetics: probe placement and control experiments. **a,** Schematic showing the placement of wireless optogenetic probes in the LEC. **b,** Stimulation did not alter total object exploration time (combined exploration of the familiar and novel objects), as yoke and driver mice showed similar exploration times (unpaired t-test, t = 2.01). **c,** Optogenetic stimulation did not affect locomotor activity, as yoke and driver mice showed similar levels of movement (unpaired t-test, t = 0.8133). **d,** Aged mice with probe implants but no light stimulation explored the familiar and novel objects to a similar extent, indicating impaired novelty discrimination (paired t-test, t = 0.2936). **e,** Discrimination index values in no-light control mice did not differ from zero (one-sample t-test, t = 0.3624). Exact p values are shown above the brackets.
